# Supplementary material for: Plasma extracellular vesicle proteomics nominates candidate biomarkers of 177Lu-PSMA-617 outcomes in metastatic prostate cancer patients
Source: Cell Rep Med. 2026 Apr 20;7(5):102764. doi: 10.1016/j.xcrm.2026.102764 (PMC13198300; doi:10.1016/j.xcrm.2026.102764)
Supplement: Document S1. Figures S1–S6 [file mmc1.pdf]

Cell Reports Medicine, Volume *7*

Supplemental information

Plasma extracellular vesicle proteomics nominates candidate biomarkers of ^{177}Lu-PSMA-617 outcomes in metastatic prostate cancer patients

Ali T. Arafa, Ella Boytim, Megan L. Ludwig, Lily Kollitz, Tianzhong Yang, Kathleen M. Storey, Stuart Bloom, Gautam Jha, Ian Okazaki, Charles J. Ryan, Nicholas A. Zorko, Daniel Steinberger, Zuzan Cayci, Yingchun Zhao, Peter W. Villalta, Scott M. Dehm, Justin H. Hwang, Justin M. Drake, and Emmanuel S. Antonarakis

Supplemental Figure 1

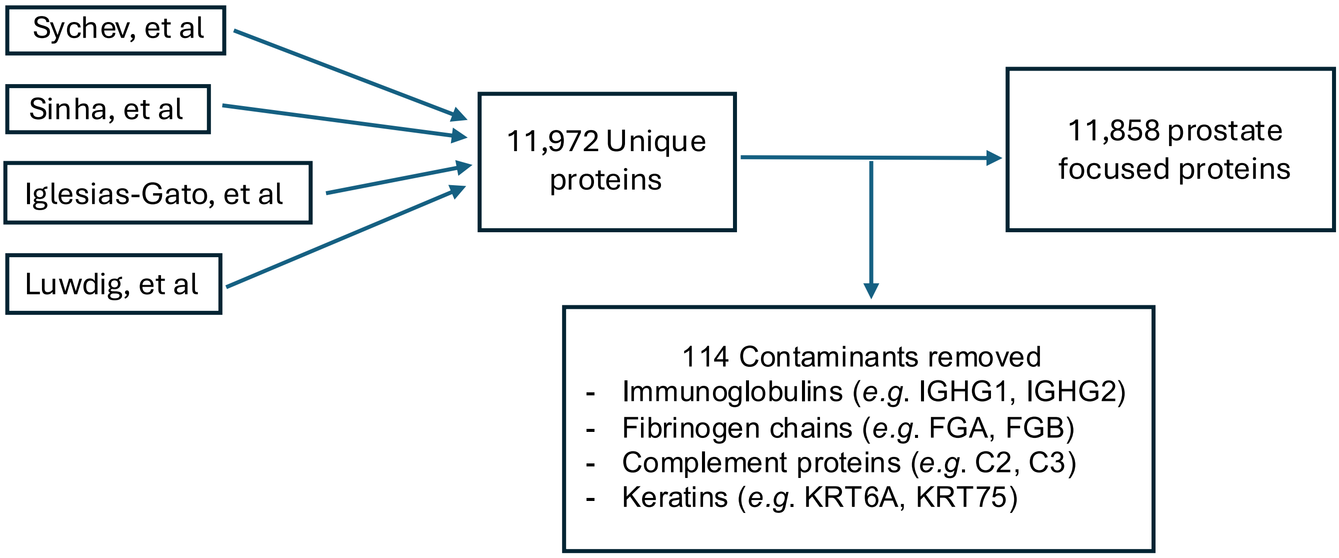

Supplemental Figure 1. Consort-like diagram summarizing how the EV proteomic library was created. A total of 11,972 proteins were identified by integrating prostatic cancer proteomic datasets from four independent studies: Sychev et al., 2024, Sinha et al., 2019, Iglesias-Gato et al., 2018, and Ludwig et al., 2025. After removing 114 contaminants, including immunoglobulins (e.g., IGHG1, IGHG2), fibrinogen chains (e.g., FGA, FGB, FGG), complement proteins (e.g., C2, C3), and keratins (e.g., KRT8, KRT17), a total of 11,858 prostate focused proteins were retained for the final EV proteomic library.

Supplemental Figure 2

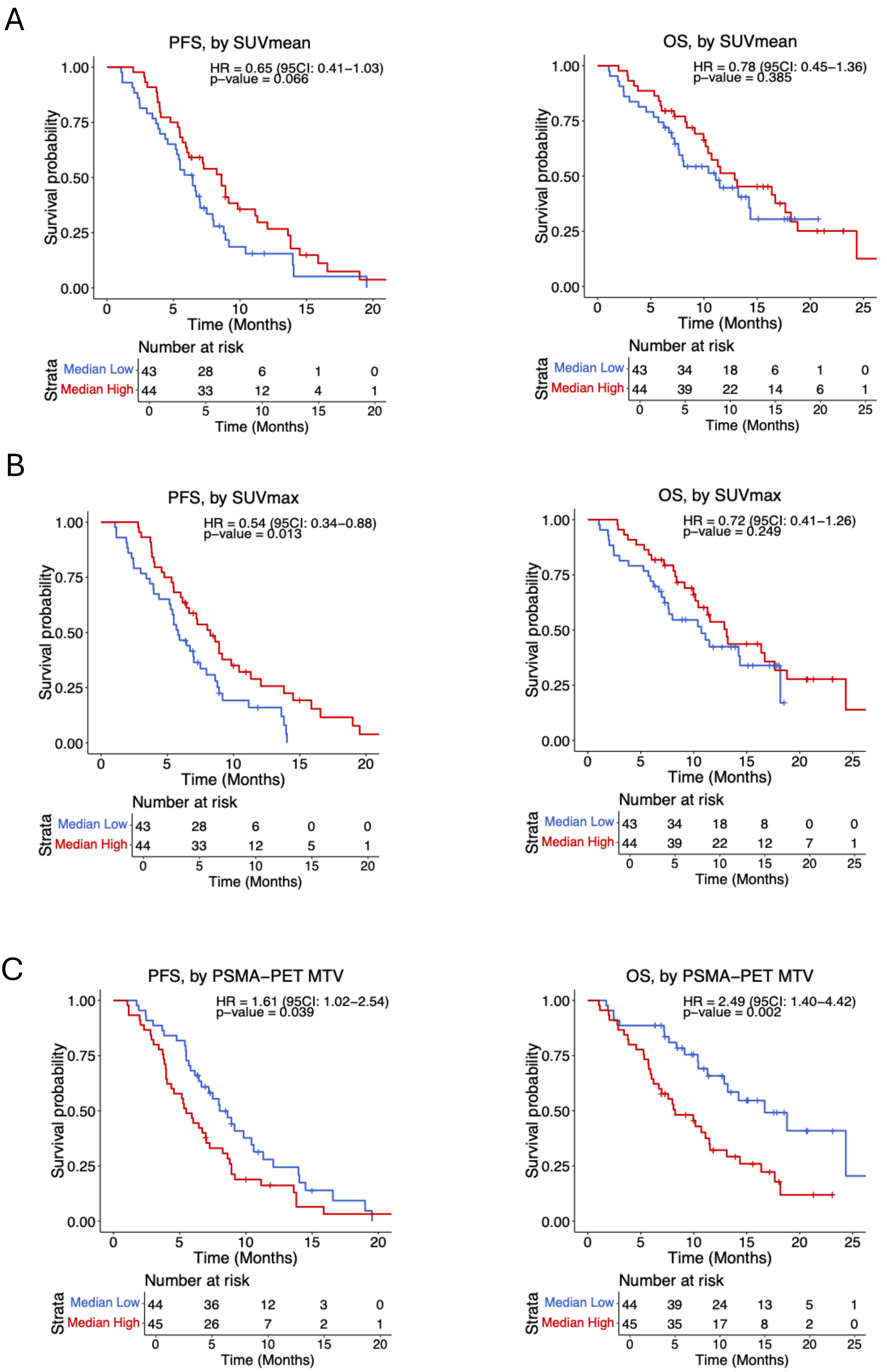

Supplemental Figure 2: Prognostic value of PSMA-PET imaging parameters in patients undergoing ^{177}Lu-PSMA-617 therapy. A. Kaplan-Meier curves showing no significant association between SUVmean and PFS (left) or OS (right). B. Kaplan-Meier curves showing that higher SUVmax is significantly associated with improved PFS (left) but not OS (right). C. Kaplan-Meier analysis of molecular tumor volume (MTV) burden showing that patients with high MTV had worse PFS (left) or OS (right).

Supplemental Figure 3

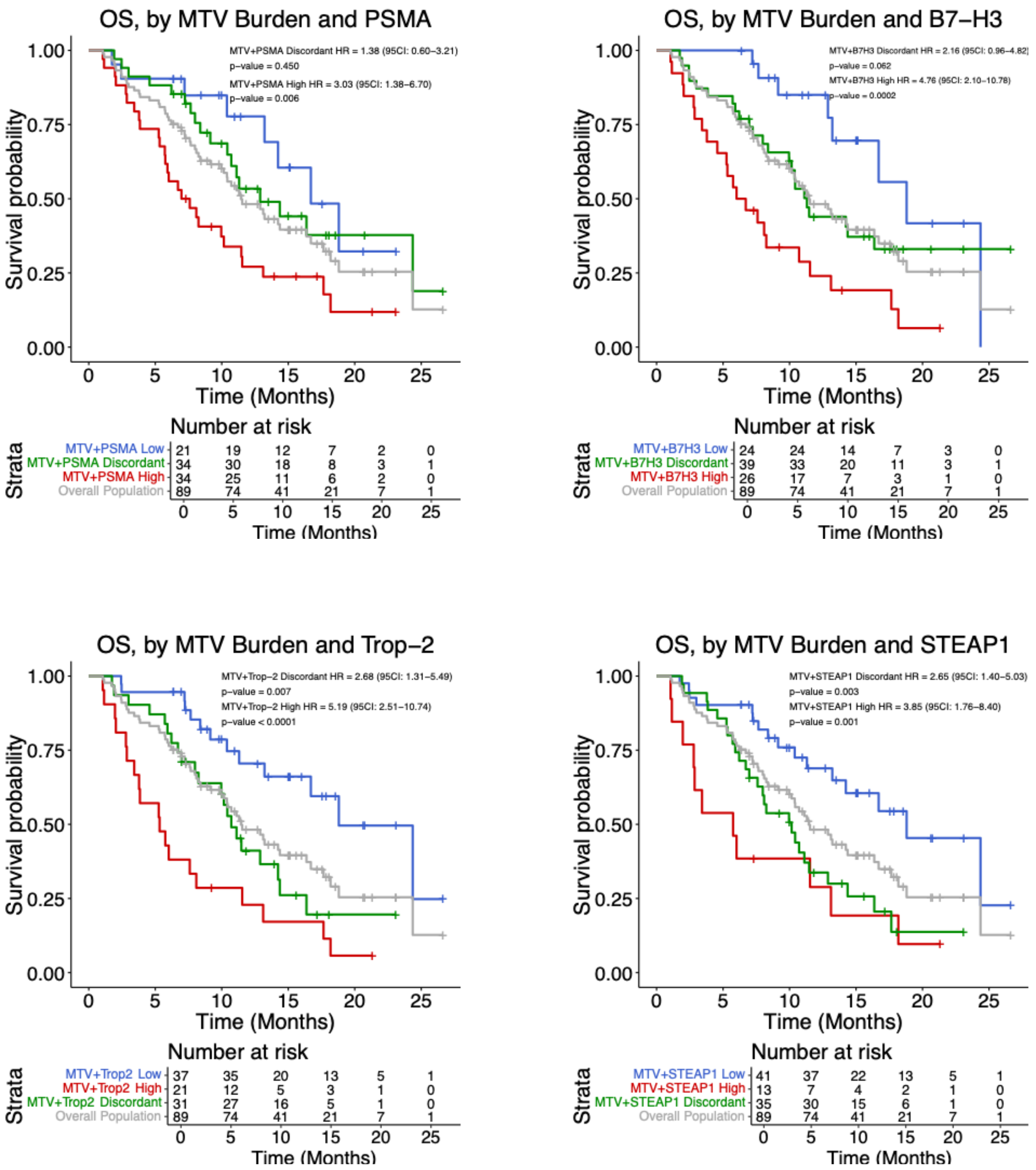

Supplemental Figure 3: Prognostic utility of EV surface proteins beyond MTV burden. A. Kaplan-Meier curve showing that MTV burden + PSMA high levels were significantly associated with worse overall survival (OS). B. Kaplan-Meier curve showing that MTV burden + B7-H3 high levels were significantly associated with worse overall survival (OS). C. Kaplan-Meier curve showing that MTV burden + Trop-2 high levels were significantly associated with worse overall survival (OS). D. Kaplan-Meier curve showing that MTV burden + STEAP1 high levels were significantly associated with worse overall survival (OS).

Supplemental Figure 4

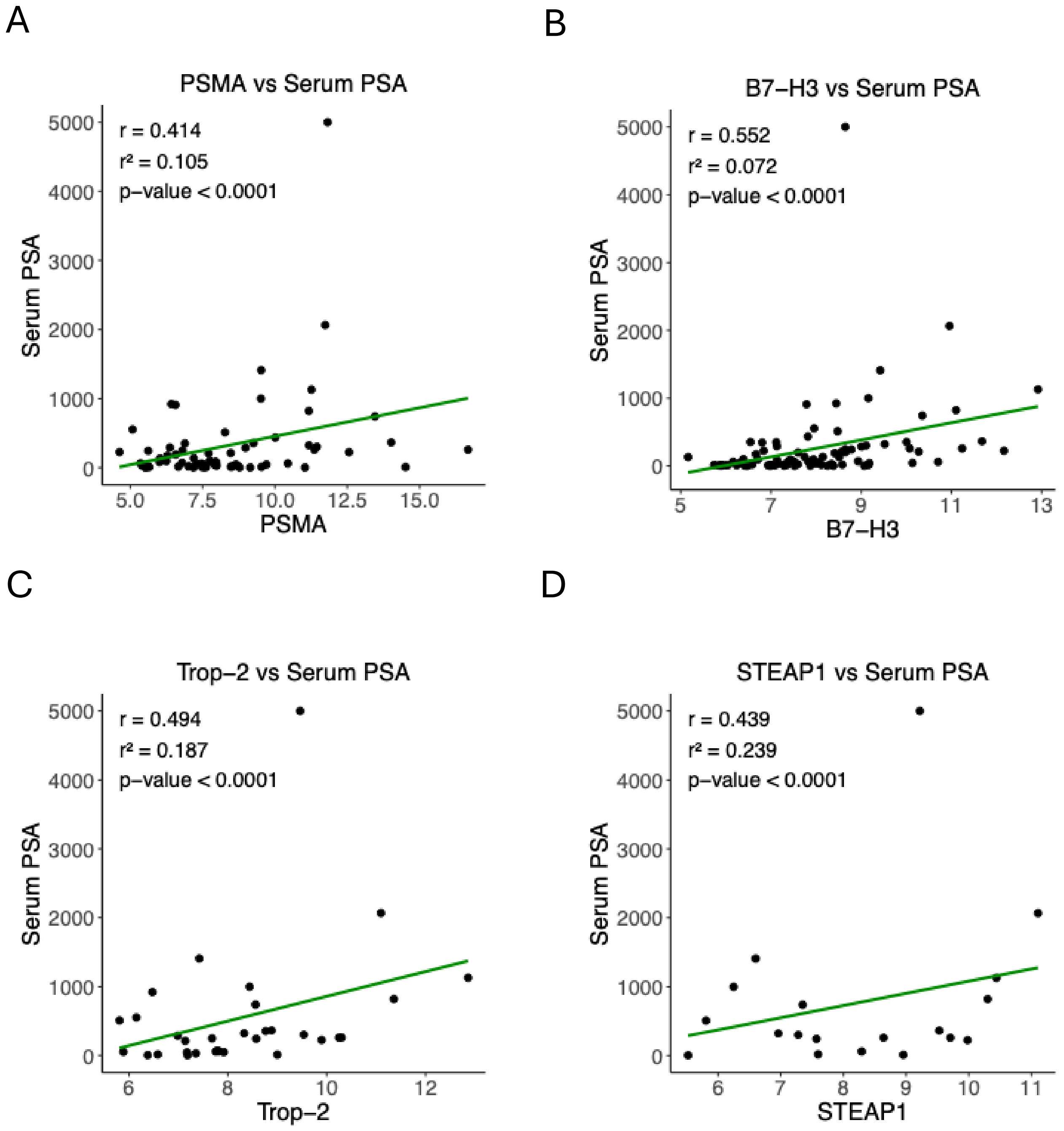

Supplemental Figure 4. Correlation between EV surface proteins and serum PSA levels. A. Correlation between serum PSA levels (y-axis) and EV-derived PSMA protein (x-axis). B. Correlation between serum PSA levels (y-axis) and EV-derived B7H3 protein (x-axis). C. Correlation between serum PSA levels (y-axis) and EV-derived Trop-2 protein (x-axis). D. Correlation between serum PSA levels (y-axis) and EV-derived STEAP1 protein (x-axis).

Supplemental Figure 5

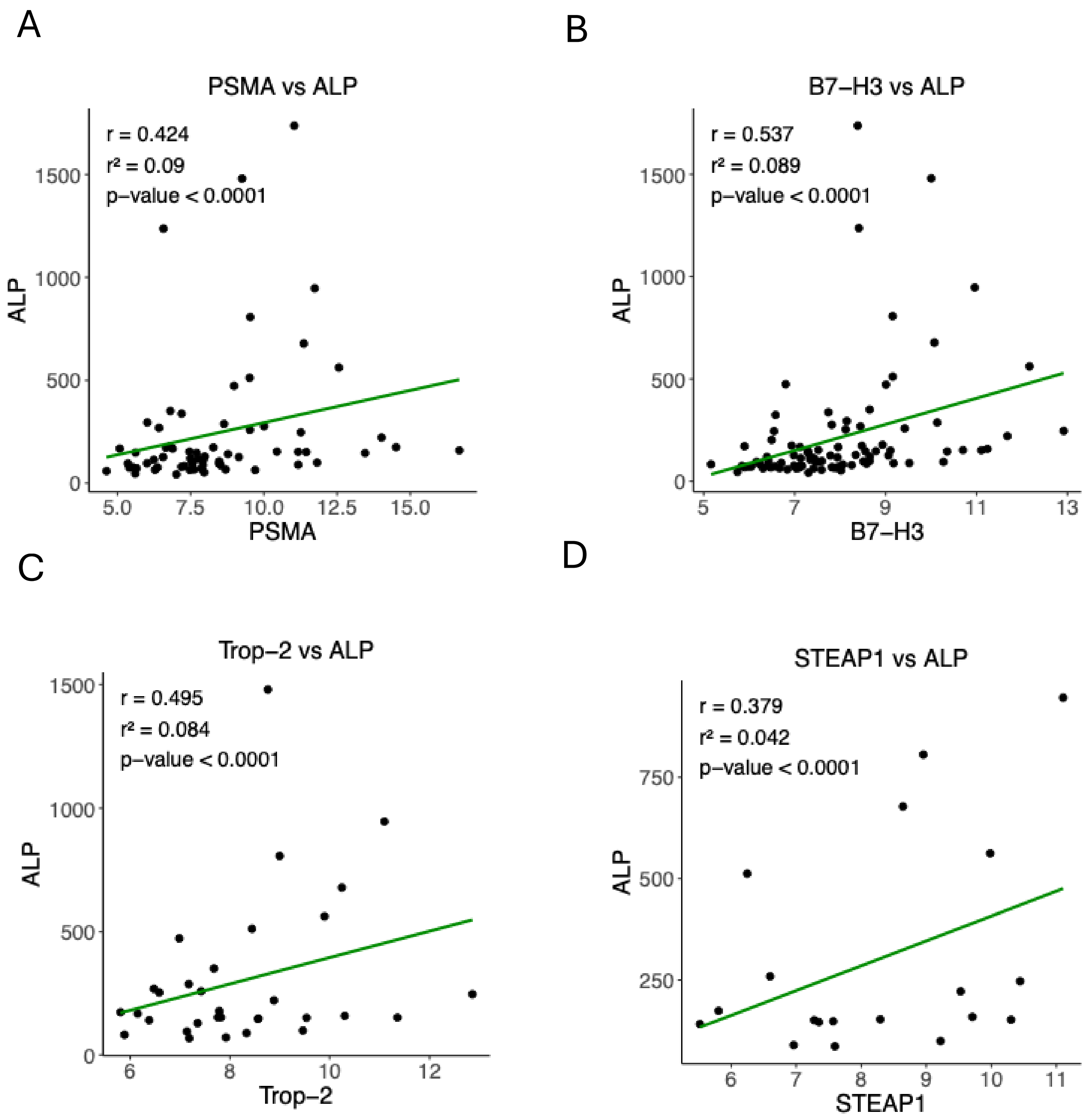

Supplemental Figure 5. Correlation between EV surface proteins and serum ALP levels. A. Correlation between serum ALP levels (y-axis) and EV-derived PSMA protein (x-axis). B. Correlation between serum ALP levels (y-axis) and EV-derived B7H3 protein (x-axis). C. Correlation between serum ALP levels (y-axis) and EV-derived Trop-2 protein (x-axis). D. Correlation between serum ALP levels(y-axis) and EV-derived STEAP1 protein (x-axis).

Supplemental Figure 6

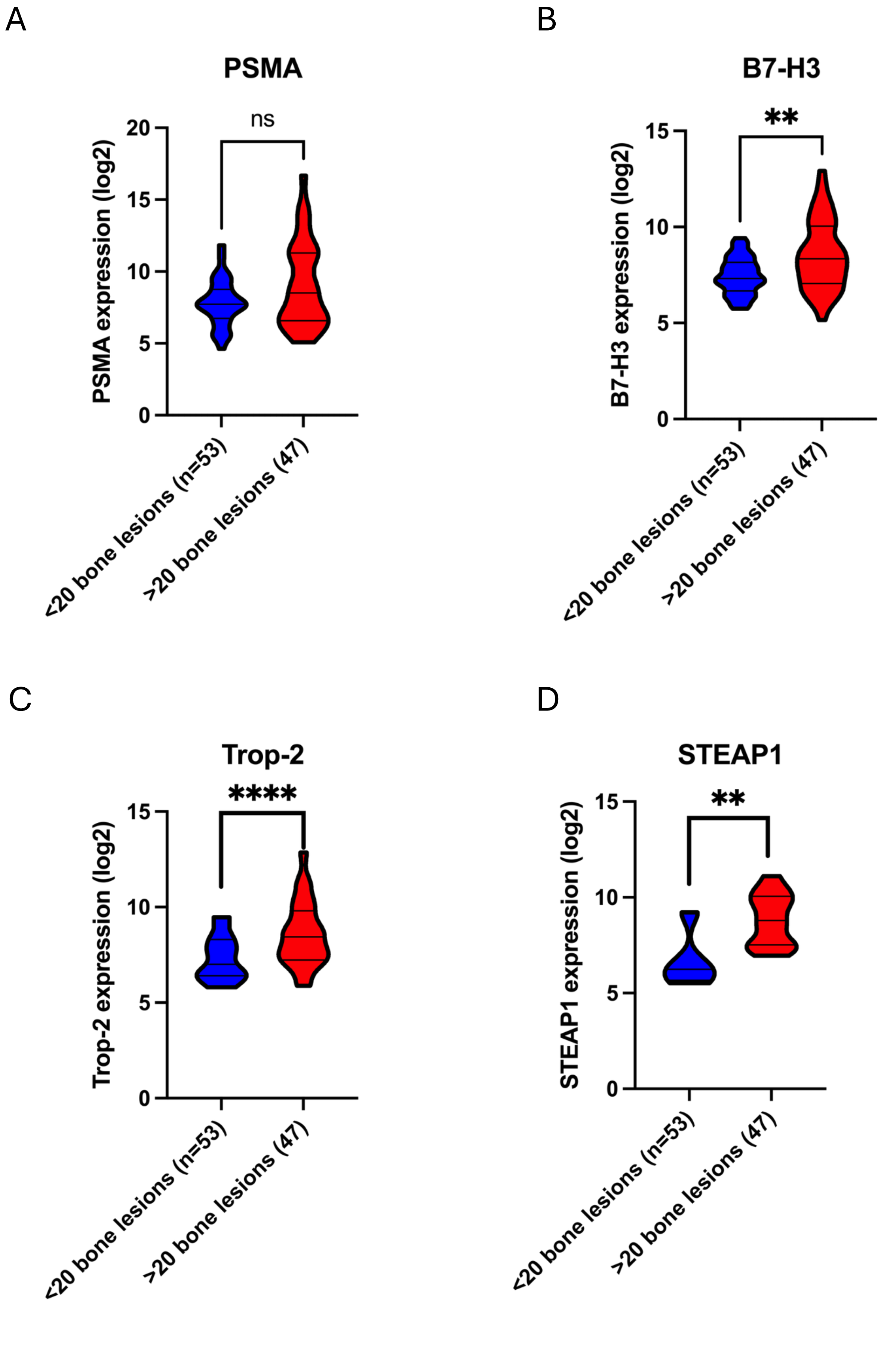

Supplemental Figure 6. EV protein expression association with bone metastatic burden. Violin plots comparing EV surface protein expression between patients with <20 vs >20 bone metastases with respect to A. PSMA protein, B. B7-H3 protein, C. Trop-2 protein, and D. STEAP1 protein. Plots display median expression levels, with x-axis indicating bone metastasis groups and y-axis representing $\log_{10}$-transformed protein expression values. Asterisk marks denote level of statistical significance (Mann Whitney U-tests) : **p* <0.05, ***p* <0.01, ****p* <0.001, *****p* <0.0001.
