# Supplementary material for: Plasma extracellular vesicle proteomics nominates candidate biomarkers of 177Lu-PSMA-617 outcomes in metastatic prostate cancer patients
Source: Cell Rep Med. 2026 Apr 20;7(5):102764. doi: 10.1016/j.xcrm.2026.102764 (PMC13198300; doi:10.1016/j.xcrm.2026.102764)
Supplement: Document S2. Article plus supplemental information [file mmc13.pdf]

Cell Reports Medicine

Article

# Plasma extracellular vesicle proteomics nominates candidate biomarkers of $^{177}$Lu-PSMA-617 outcomes in metastatic prostate cancer patients

## Graphical abstract

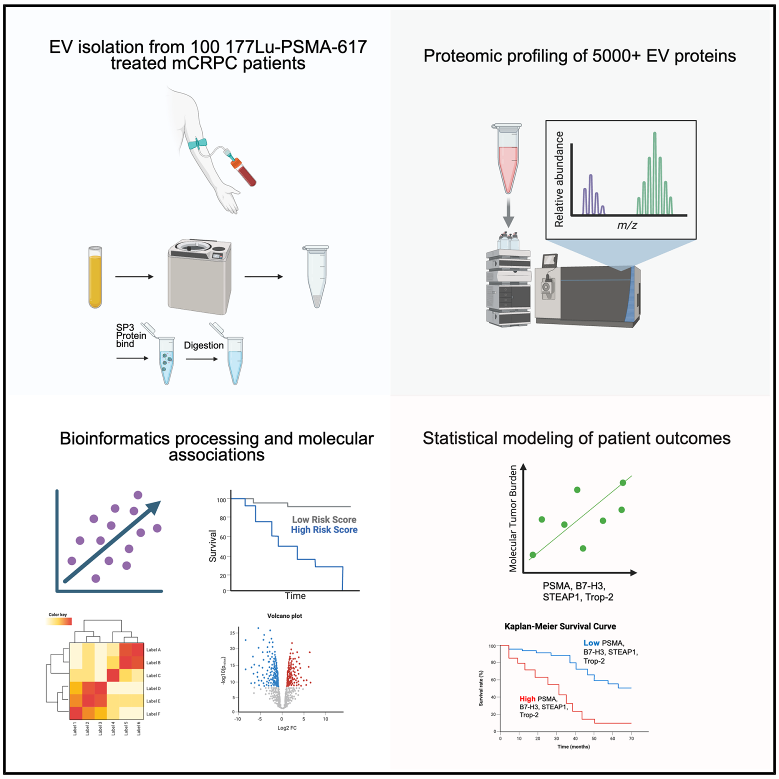


## Highlights

- Shotgun proteomics of plasma EVs identifies 5,137 proteins in mCRPC patients
- EV-derived PSMA, B7-H3, Trop-2, and STEAP1 associate with worse OS
- PSMA+ and PSMA− CTC subpopulations predict worse overall survival
- p53 upregulation confers resistance; activated E2F pathway portends better outcomes


## Authors

Ali T. Arafa, Ella Boytim, Megan L. Ludwig, ..., Justin H. Hwang, Justin M. Drake, Emmanuel S. Antonarakis

## Correspondence

jdrake@umn.edu (J.M.D.), anton401@umn.edu (E.S.A.)



## In brief

Arafa et al. profile plasma extracellular vesicle (EV) proteomes from 100 metastatic castration-resistant prostate cancer patients receiving $^{177}$Lu-PSMA-617. EV-derived PSMA, B7-H3, Trop-2, and STEAP1 are associated with worse survival. PSMA-positive and PSMA-negative CTC subpopulations and EV pathway signatures reveal distinct tumor biology linked to treatment outcomes.


Arafa et al., 2026, Cell Reports Medicine 7, 102764
May 19, 2026 © 2026 The Author(s). Published by Elsevier Inc.
https://doi.org/10.1016/j.xcrm.2026.102764

CellPress

Cell Reports Medicine

CellPress
OPEN ACCESS

Article

# Plasma extracellular vesicle proteomics nominates candidate biomarkers of $^{177}$Lu-PSMA-617 outcomes in metastatic prostate cancer patients

Ali T. Arafa,[1] Ella Boytim,[2] Megan L. Ludwig,[1] Lily Kollitz,[3] Tianzhong Yang,[4] Kathleen M. Storey,[2] Stuart Bloom,[2] Gautam Jha,[2] Ian Okazaki,[2] Charles J. Ryan,[5] Nicholas A. Zorko,[2] Daniel Steinberger,[3] Zuzan Cayci,[3] Yingchun Zhao,[6] Peter W. Villalta,[6,7] Scott M. Dehm,[6,8,9] Justin H. Hwang,[2,6] Justin M. Drake,[1,6,9,10,*] and Emmanuel S. Antonarakis[2,6,11,*]

[1]Department of Pharmacology, University of Minnesota, Minneapolis, MN 55455, USA
[2]Division of Hematology, Oncology and Transplantation, Department of Medicine, University of Minnesota, Minneapolis, MN 55455, USA
[3]Nuclear Medicine Division, Department of Radiology, University of Minnesota Medical School, Minneapolis, MN 55455, USA
[4]Division of Biostatistics and Health Data Science, School of Public Health, University of Minnesota, Minneapolis, MN, USA
[5]Memorial Sloan Kettering Cancer Center, New York, NY 10065, USA
[6]Masonic Cancer Center, University of Minnesota, Minneapolis, MN 55455, USA
[7]Department of Medicinal Chemistry, University of Minnesota, Minneapolis, MN 55455, USA
[8]Department of Laboratory Medicine and Pathology, University of Minnesota, Minneapolis, MN 55455, USA
[9]Department of Urology, University of Minnesota, Minneapolis, MN 55455, USA
[10]Astrin Biosciences, Saint Paul, MN 55114, USA
[11]Lead contact
*Correspondence: jdrake@umn.edu (J.M.D.), anton401@umn.edu (E.S.A.)
https://doi.org/10.1016/j.xcrm.2026.102764

## SUMMARY

$^{177}$Lu-PSMA-617 represents a transformative treatment for metastatic castration-resistant prostate cancer (mCRPC), yet biomarkers of benefit beyond prostate-specific membrane antigen positron emission tomography (PSMA-PET) imaging remain lacking. Here, we present findings from a prospective study of 100 mCRPC patients receiving $^{177}$Lu-PSMA-617, using shotgun proteomics to profile plasma-derived extracellular vesicle (EV) proteins alongside PSMA-positive circulating tumor cell (CTC) enumeration. We identify 5,137 EV-derived proteins, including the cell-surface targets PSMA, B7-H3, Trop-2, and STEAP1, with high levels of these proteins associating with worse overall survival (OS). All four EV proteins positively correlate with molecular tumor volume on PSMA-PET imaging, serum PSA, and serum alkaline phosphatase. CTC subpopulations, including PSMA+/EpCAM+ and PSMA−/EpCAM+ cells, associate with worse progression-free survival (PFS) and OS. Pathway analysis reveals that p53 upregulation associates with poor PFS and OS, while an activated E2F pathway unexpectedly portends better PFS and OS. These findings support the integration of liquid biopsy proteomics into biomarker-driven mCRPC trials to improve patient stratification.

## INTRODUCTION

$^{177}$Lu-PSMA-617 has redefined the therapeutic landscape of metastatic castration-resistant prostate cancer (mCRPC), introducing a new class of protein-targeted therapies distinct from traditional hormone therapies and chemotherapies that have long served as the standard of care for advanced disease.[1] By linking a PSMA-617 ligand that binds prostate-specific membrane antigen (PSMA) to the beta-emitting lutetium-177 isotope, this therapy delivers DNA-damaging radiation to PSMA-expressing tumor cells, while generally sparing surrounding healthy tissue.[2] Clinical trials including VISION, PSMAfore, and PSMAddition have demonstrated that $^{177}$Lu-PSMA-617 significantly improves clinical outcomes, establishing its role across multiple disease stages.[3–5] Despite this breakthrough, therapeutic responses to $^{177}$Lu-PSMA-617 remain heterogeneous, with a subset of patients showing primary resistance.[6] Therefore, there is an unmet need to identify biomarkers that may predict therapeutic efficacy and guide patient selection beyond the use of PSMA-PET (positron emission tomography) imaging parameters alone.[7,8]

While certain genomic alterations have been linked to resistance to radiation therapies, including $^{177}$Lu-PSMA-617, the drivers of disease remain unknown.[9,10] This gap is critical given the rise of protein-targeting agents in the prostate cancer therapeutic landscape, including radioligand therapies, antibody-drug conjugates (ADCs), and bi-specific T cell engagers exploiting cell-surface markers such as PSMA, B7-H3, Trop-2, and STEAP1.[11–14] However, quantifying these proteins in circulation has been very challenging given the abundance of circulating plasma proteins such as albumin and immunoglobulins that obscure the detection of tumor-derived markers.[15]

Plasma extracellular vesicles (EVs) represent a promising liquid biopsy approach to overcome limitations of current


© 2026 The Author(s). Published by Elsevier Inc.
This is an open access article under the CC BY license (http://creativecommons.org/licenses/by/4.0/).

blood-based biomarkers.[16] These membranous nanoparticles, secreted by all cells (including tumor cells), carry proteins, lipids, and nucleic acids that reflect the molecular state of their cell of origin.[17,18] Recent advances in EV isolation, particularly through differential ultracentrifugation, combined with improvements in mass spectrometry sensitivity coupled with data-independent acquisition mass spectrometry (DIA-MS), have gained traction for their ability to comprehensively quantify proteins across a dynamic range.[19,20] Unlike conventional data-dependent acquisition methods, which selectively fragment the most abundant peptides, DIA methods systematically fragment all peptides within defined mass windows, capturing a more complete and unbiased proteomic profile. This approach improves sensitivity for low-abundance proteins and enhances reproducibility across samples, making it particularly well-suited for complex, low-input sources such as plasma-derived EVs.[21,22]

In addition, circulating tumor cells (CTCs) represent an alternative liquid biopsy modality for noninvasive cancer detection, prognostication, and treatment monitoring of mCRPC patients.[23–26] Recent advances, including the integration of inertial microfluidics, digital holographic microscopy, and immunofluorescence, have enabled high-throughput label-free CTC detection with improved sensitivity and specificity.[27]

Here, we hypothesized that deep proteomic analysis of plasma EVs using unbiased DIA-MS-based proteomics could uncover biomarkers of sensitivity and resistance in patients receiving $^{177}$Lu-PSMA-617. To test this, we conducted a prospective biomarker study of 100 mCRPC patients. We interrogated key cell-surface targets including PSMA, B7-H3, Trop-2, and STEAP1; identified tumor-enriched proteins and pathways; enumerated PSMA-positive CTC subpopulations; and tested the clinical utility of these biomarkers for predicting outcomes to $^{177}$Lu-PSMA-617 treatment.

## RESULTS

### Patient cohort

One hundred mCRPC patients were prospectively enrolled prior to treatment with $^{177}$Lu-PSMA-617 (Figure 1). Baseline patient characteristics are summarized in Table 1. We also enrolled 20 cancer-free male controls with a median age of 60 (range, 54–75) years. The median age at enrollment of the 100 mCRPC patients was 74 years (range, 44–94), and the median baseline PSA level was 54.5 ng/mL (range, 1.42–5,000). Gleason grade groups (GGs) 1–3 were observed in 16% of patients, GG4 in 16%, and GG5 in 47%, with 21% of patients having unknown Gleason grade. The majority of patients had bone metastases (91%), 63% showed lymph node involvement, and 12% had visceral (liver and lung) metastasis. The median baseline molecular tumor volume (MTV) was 91.2 cm$^3$ (range, 2.00–983.06), with PSMA-PET standardized uptake value mean (SUVmean) and standardized uptake value max (SUVmax) of 7.97 (range, 1.52–61.78) and 33.4 (range, 8.42–401.64), respectively. Eastern Cooperative Oncology Group performance status of 0–1 was seen in 79% of patients. The majority of patients (72%) had received three or more life-prolonging systemic therapies, including androgen receptor pathway inhibitors (abiraterone 67%, enzalutamide 62%, apalutamide 11%, darolutamide 15%) and chemotherapy agents (docetaxel 85%, cabazitaxel 30%). Among the treated mCRPC cohort, the median PFS was 212 (range, 32–648) days, while the PSA50 response rate was 44%. Patients received between 1 and 6 administered doses, with 13% receiving 1 dose, 14% receiving 2 doses, 12% receiving 3 doses, 16% receiving 4 doses, 7% of patients receiving 5 doses, and 38% receiving all 6 doses.

### PSMA-PET imaging parameters and outcomes

We investigated standard PSMA-PET parameters that are sometimes used to aid clinical decision-making, which may impact outcomes.[28,29] In our cohort, higher baseline SUVmean was not associated with greater PFS (hazard ratio [HR] = 0.65, 95% confidence interval [CI]: 0.41–1.03, $p$ = 0.066) or overall survival [OS] (HR = 0.78, 95% CI: 0.45–1.36, $p$ = 0.385) (Figure S2A), although suggestive trends were seen. Higher SUVmax was associated with improved PFS (HR = 0.54, CI: 0.34–0.88, $p$ = 0.013), but not OS (HR = 0.72, 95% CI: 0.41–1.26, $p$ = 0.249) (Figure S2B). We then investigated the impact of MTV burden on patients receiving $^{177}$Lu-PSMA-617. In our cohort, high MTV burden was associated with worse PFS (HR = 1.61, 95% CI: 1.02–2.54, $p$ = 0.039) and predicted significantly worse OS (HR = 2.49, 95% CI 1.40–4.42, $p$ = 0.002) (Figure S2C). While MTV burden was the best imaging parameter at predicting survival outcomes, it remains impractical for patients with extensive metastases due to the requirement for labor-intensive manual segmentation. These constraints highlight the need for minimally invasive biomarkers such as plasma EV proteomics to help improve clinical decision-making.

### Characterization of EV-derived proteins

We evaluated the robustness of EV detection across all 100 patients by examining the size distribution of baseline EVs isolated from plasma, measured by nanoparticle tracking analysis (NTA). The majority of detected particles were between 100 and 200 nm, consistent with the expected size of small EVs isolated using differential ultracentrifugation (Figure 2A). Next, we performed deep proteomic interrogation of these plasma EVs using unbiased DIA-MS-based proteomics. A total of 5,137 EV-derived proteins were identified across the cohort (Table S1), with a median of 3,767 proteins detected per patient (range, 2,519–4,555) (Figure 2B). To evaluate assay reproducibility, we conducted technical duplicate injections on 20 randomly selected samples. Principal-component analysis showed that each set of technical replicates clustered with each other consistently (Figure 2C), while pairwise correlation analysis revealed correlations of >94%, confirming the reproducibility of our runs. We then examined several canonical EV markers and grouped them by functional class, including tetraspanins, scaffold proteins, lipid raft-associated proteins, and trafficking/secretion regulators. These EV-associated markers were detected across the cohort, supporting the consistency of our EV purification and proteomics workflow (Figure 2D). To further characterize the EV proteins detected, we considered their annotated subcellular localizations. Among the 5,137 proteins identified, 18.8% were predominantly localized to the cell membrane, 19.5% to the nucleus, 36.4% to the cytoplasm, 9.6% to mitochondria, and 15.7% to other compartments including the endoplasmic reticulum, endosomes, Golgi apparatus, peroxisomes, and lysosomes (Figure 2E).

**Table 1. Baseline characteristics of mCRPC patients enrolled in this study**

| Number of enrolled mCRPC patients | 100 (100%) |
|---|---|
| Age at diagnosis (years) | |
| Median (min–max) | 66 (42–90) |
| Age at study enrollment (years) | |
| Median (min–max) | 74 (44–94) |
| Race | |
| White | 92 (92%) |
| Black/Asian | 8 (8%) |
| PSA level at diagnosis (ng/mL) | |
| Median (min–max) | 44 (1.7–8,970) |
| Gleason grade group (%) | |
| 1–3 | 16 (16%) |
| 4 | 16 (16%) |
| 5 | 47 (47%) |
| Unknown | 21 (21%) |
| Baseline serum PSA (ng/mL) | |
| Median (min–max) | 54.5 (1.42 – 5,000) |
| Baseline serum ALP levels (U/L) | |
| Median (min–max) | 109 (42–1,736) |
| Baseline serum Hb levels (g/dL) | |
| Median (min–max) | 11.65 (7.60–16.30) |
| Baseline serum Alb levels (g/dL) | |
| Median (min–max) | 3.9 (2.70–4.80) |
| Baseline MTV on PSMA-PET imaging ($cm^3$) | |
| Median (min–max) | 91.2 (2.0–983.1) |
| Baseline PSMA-PET SUVmean | |
| Median (min–max) | 7.9 (1.5–61.8) |
| Baseline PSMA-PET SUVmax | |
| Median (min–max) | 33.4 (8.4–401.6) |
| Baseline ECOG performance status | |
| 0 | 24 (24%) |
| 1 | 55 (55%) |
| 2–3 | 21 (21%) |
| Site of distant metastasis at baseline: no. (%) | |
| Bone | 91 (91%) |
| Lymph node | 63 (63%) |
| Visceral | 12 (12%) |
| Previous local therapies: no. (%) | |
| Radical prostatectomy | 25 (25%) |
| Primary radiation therapy | 39 (39%) |
| Number of prior systemic therapies received | |
| 1–2 | 28 (28%) |
| 3 or more | 72 (72%) |
| Specific prior systemic therapies: no. (%) | |
| ARPIs | |
| Abiraterone | 67 (67%) |
| Enzalutamide | 62 (62%) |
| Apalutamide | 11 (11%) |
| Darolutamide | 15 (15%) |
| Chemotherapy | |
| Docetaxel | 85 (85%) |
| Cabazitaxel | 30 (30%) |
| Carboplatin | 13 (13%) |
| Other | |
| PARP inhibitor | 9 (9%) |
| Pembrolizumab | 3 (3%) |
| Radium-223 | 2 (2%) |

mCRPC, metastatic castrate-resistant prostate cancer; EV, extracellular vesicles; CTC, circulating tumor cells; PSA, prostate specific antigen; ALP, alkaline phosphatase; Hb, hemoglobin; Alb, albumin; PSMA-PET, prostate-specific membrane antigen positron emission tomography; SUV, standardized uptake value; MTV, molecular tumor volume; ECOG, Eastern Cooperative Oncology Group; PARP, poly(ADP-ribose) polymerase; ARPI, androgen receptor pathway inhibitor.

To evaluate the prognostic significance of detecting cell-surface proteins that are relevant in prostate cancer, we stratified patients based on the presence or absence of four key CRPC-associated proteins currently being investigated in clinical trials for advanced prostate cancer patients: PSMA, B7-H3, Trop-2, and STEAP1. Patients were stratified into three groups: 0–1 detected proteins, 2 detected proteins, and 3–4 detected proteins. Kaplan-Meier survival analysis showed a progressive decline in OS with an increasing number of detected proteins (Figure 2F). This suggests that patients with detection of 3–4 of these proteins of interest exhibit a broader tumor-associated EV protein profile compared to patients with detection of 0–1 of these proteins. We next assessed whether an increased number of detectable EV-derived cell-surface proteins was associated with PSMA-PET tumor burden. Using the same stratification groups, we observed a significant increase in MTV burden with higher numbers of detected proteins (Figure 2G). Again, these findings support the notion that the detection of these four CRPC-associated proteins likely reflects tumor-derived EV secretion.

## Prognostic significance of specific EV proteins

To identify prognostic biomarkers of $^{177}$Lu-PSMA-617 sensitivity or resistance, we assessed the association between EV surface proteins and clinical outcomes. In our plasma EV assay, PSMA, B7-H3, Trop-2, and STEAP1 were detected in 66%, 91%, 32%, and 19% of patients, respectively (Table S2). In the cancer-free healthy-control cohort, PSMA, B7-H3, and STEAP1 were not detected, while 10% of patients had detectable Trop-2 levels (Figure 1; Table S3). Detection of EV-derived PSMA protein was associated with significantly shorter OS (HR = 1.80, 95% CI: 1.02–3.19, $p$ = 0.043), but not PFS (HR = 1.18, 95% CI: 0.74–1.87, $p$ = 0.494). PSMA protein concentration also correlated with MTV burden (r = 0.328, $p$ = 0.002) (Figure 3A). STEAP1 was associated with significantly shorter OS (HR = 2.99, 95% CI: 1.70–5.25, $p$ = 0.001), but not PFS (HR = 1.59, 95% CI: 0.94–2.69, $p$ = 0.082). STEAP1 concentration also significantly

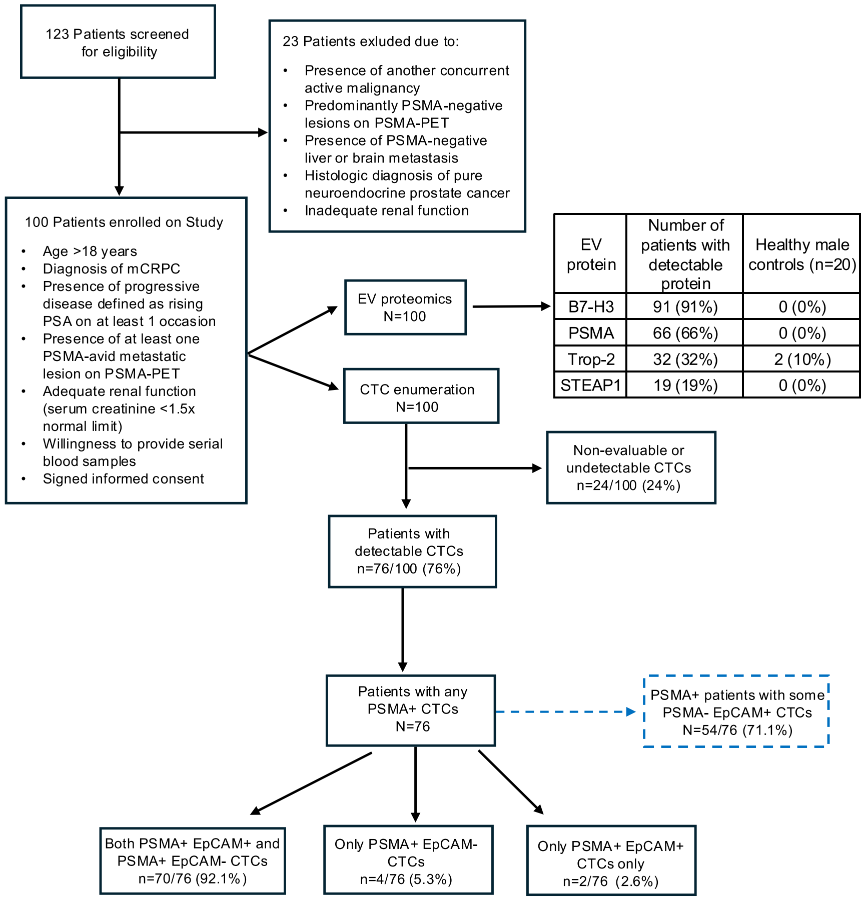


**Figure 1. Consort diagram summarizing patient enrollment and EV/CTC profiling**

A total of 100 patients were prospectively enrolled and underwent blood-based extracellular vesicle (EV) proteomic analysis. PSMA, B7-H3, Trop-2, and STEAP1 proteins were detected in 66%, 91%, 32%, and 19% of patients, respectively. Circulating tumor cell (CTC) enumeration was performed in 76 evaluable patients, all of whom (100%) had detectable PSMA+ CTCs. Upon further stratification, 70/76 (92.1%) harbored both PSMA+ EpCAM+ and PSMA+ EpCAM− CTCs, 4/76 (5.3%) had only PSMA+ EpCAM− CTCs, and 2/76 (2.6%) had only PSMA+ EpCAM+ CTCs. In addition, 54/76 patients (71.1%) had some detectable PSMA− EpCAM+ CTCs in their pooled cell population.

correlated with MTV burden (r = 0.358, $p$ = 0.0006) (Figure 3D). B7-H3 protein was significantly associated with worse PFS (HR = 1.86, 95% CI: 1.22–285, $p$ = 0.004) as well as OS (HR = 2.51, 95% CI: 1.50–4.21, $p$ = 0.0004). A positive correlation with baseline MTV burden was also observed (r = 0.459, $p < 0.0001$) (Figure 3B). Finally, detectable Trop-2 protein was associated with shorter PFS (HR = 1.59, 95% CI: 1.37–3.34, $p$ = 0.001) and OS (HR = 3.71, 95% CI: 2.22–6.20, $p < 0.0001$) and also positively correlated with MTV burden (r = 0.431, $p < 0.0001$) (Figure 3C).

We next expanded our analysis to a broader panel of known as well as EV-derived surface proteins. Elevated levels of TMPRSS2, HER2 (ERBB2), and STEAP1B were significantly associated with both shorter PFS and OS, suggesting their potential role as markers of aggressive disease biology. In contrast, higher expression of CNTN1, CD46, CD151, and CXCR4 were associated with improved OS (Figures 3E and 3F) (Table S4). These findings nominate a subset of EV proteins that may inform future drug development in mCRPC patients or serve as stratification biomarkers in prospective clinical trials.

## High MTV burden and higher levels of EVs predict worse survival outcomes

To examine whether the prognostic associations of individual EV proteins were independent of baseline disease burden, we performed stratified survival analyses combining MTV burden with tumor-associated EV protein detection (Figure S3). High-MTV plus high-PSMA expression was associated with significantly worse OS (HR = 3.03, 95% CI: 1.38–6.76, $p$ = 0.006) compared with low-MTV/low-PSMA patients. High-MTV plus high B7-H3 was also linked with inferior OS (HR = 4.76, 95% CI: 2.10–10.78, $p$ = 0.0002). Similarly, high MTV plus high Trop-2 expression showed significantly worse OS (HR = 5.19, 95% CI: 2.51–10.74, $p < 0.0001$), highlighting Trop-2 as a particularly strong marker of adverse outcome when combined with high tumor burden. Finally, high MTV plus high STEAP1 was also associated with poorer OS (HR = 3.85, 95% CI: 1.76–8.40, $p$ = 0.001) (Figure S3).

## EV cell-surface proteins correlate with serum biomarkers and metastatic burden

We next assessed the relationship between EV cell-surface protein expression and conventional clinical biomarkers. Across the cohort, EV-derived concentrations of PSMA (r = 0.414, $p < 0.0001$), B7-H3 (r = 0.552, $p < 0.0001$), Trop-2 (r = 0.494, $p < 0.0001$), and STEAP1 (r = 0.439, $p < 0.0001$) were positively correlated with serum PSA levels (Figure S4). Similarly, EV levels of PSMA (r = 0.424, $p < 0.0001$), B7-H3 (r = 0.537, $p < 0.0001$), Trop-2 (r = 0.495, $p < 0.0001$), and STEAP1 (r = 0.379, $p$ = 0.0001) were positively correlated with serum ALP levels (Figure S5).

To further explore the relationship between EV protein expression and molecular tumor burden, we stratified patients based on the number of bone lesions detected on PSMA-PET scan (<20 vs. > 20 lesions). EV expression of B7-H3 ($p < 0.001$), Trop-2 ($p < 0.0001$), and STEAP1 ($p < 0.01$) were significantly higher in patients with >20 bone metastases (compared to those with <20 metastases), while PSMA expression did not differ significantly between the two groups ($p$ = 0.270) (Figure S6). These findings indicate that B7-H3 and Trop-2 are enriched in high-burden osseous metastatic

CellPress
OPEN ACCESS

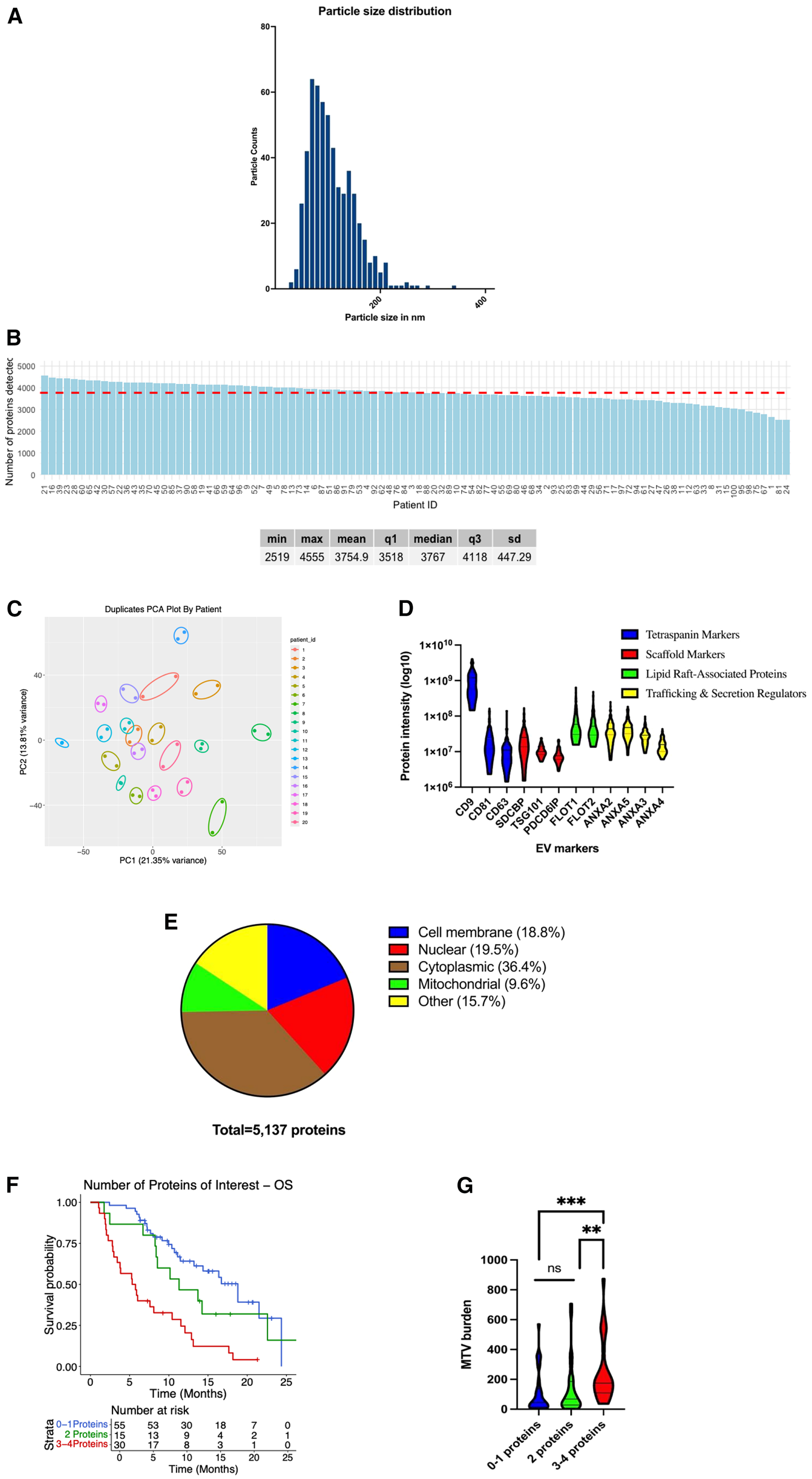


(legend on next page)

disease, supporting their relevance as targets for emerging protein-directed therapies.

## Pathway-level analysis of EV proteomes reveals prognostic signatures

To uncover potential protein pathways associated with clinical outcomes, we applied single-sample gene set enrichment analysis (ssGSEA) to EV proteomic data for all patients. The resulting heatmap revealed distinct clustering of patients based on enrichment of GSEA hallmark pathways (Figure 4A). Notably, patients in Clusters 1 and 2 showed upregulation of several hallmark pathways including EMT, adipogenesis, and the p53 pathway compared to patients in Clusters 3 and 4. This stratification suggests that EV proteomic-derived pathway signatures may reflect distinct tumor phenotypes, which would help to uncover biological mechanisms that predict treatment benefit.

Among the most strongly associated pathways, higher levels of canonical p53 pathway were associated with worse PFS (HR = 1.85, 95% CI: 1.19–2.88, $p$ = 0.006) as well as OS (HR = 2.10, 95% CI: 1.24–3.55, $p$ = 0.006). Interferon-gamma response was also associated with worse PFS (HR = 1.62, 95% CI: 1.05–2.50, $p$ = 0.030) and OS (HR = 1.82, 95% CI: 1.11–3.10, $p$ = 0.019). Increased IL6-JAK-STAT3 signaling was also linked to worse PFS (HR = 1.61, 95% CI: 1.04–2.48, $p$ = 0.032) and OS (HR = 1.85, 95% CI: 1.11–3.10, $p$ = 0.019). Additional pathways associated with poor OS included late estrogen response, MYC targets, and TGF-$\beta$ signaling. Conversely, high levels of the E2F pathway were unexpectedly linked to significantly improved PFS (HR = 0.54, 95% CI: 0.35–0.84, $p$ = 0.006) and OS (HR = 0.48, 95% CI: 0.29–0.80, $p$ = 0.005) (Figures 4B and 4C; Table S5). Together, these findings suggest that EV proteomics can capture protein signaling pathways with prognostic relevance in mCRPC patients that may indicate sensitivity or resistance to $^{177}$Lu-PSMA-617 therapy.

## CTC phenotypes reveal subsets linked to worse survival

Next, we determined whether CTC-derived PSMA expression may also serve as a prognostic marker in the context of $^{177}$Lu-PSMA-617 therapy. All patients with detectable CTCs expressed PSMA on immunohistochemical staining in at least a fraction of cells, with 70/76 (92.1%) patients expressing both PSMA+/EpCAM+ and PSMA+/EpCAM− CTC subpopulations in the same blood sample, 4/76 (5.3%) patients only expressing PSMA+/EpCAM− CTCs per blood sample, and 2/76 (2.6%) patients expressing only PSMA+/EpCAM+ CTCs. Interestingly, 54/76 (71.1%) of patients who expressed some PSMA+ CTCs also expressed a fraction of PSMA−/EpCAM+ CTCs in the same blood sample (Figures 1 and 5A; Table S2).

Higher total CTC counts, independent of PSMA or EpCAM expression, were associated with worse PFS (HR = 1.65, CI: 1.00–2.72, $p$ = 0.048) and OS (HR = 3.16, CI: 1.64–6.09, $p$ = 0.001) and were positively correlated with baseline MTV burden (r = 0.345, $p$ = 0.004) (Figure 5B). Higher PSMA+/EpCAM+ CTC counts were associated with worse PFS (HR = 2.03, CI: 1.21–3.39, $p$ = 0.007) and OS (HR = 3.79, CI: 1.90–7.56, $p$ = 0.0002) and also showed a positive correlation with MTV burden (r = 0.29, $p$ = 0.017) (Figure 5C). PSMA+/EpCAM− CTCs were significantly associated with worse OS (HR = 1.87, CI: 1.01–3.47, $p$ = 0.048) but not PFS (HR = 1.57, CI: 0.95–2.59, $p$ = 0.075). PSMA+/EpCAM− CTCs also significantly correlated with MTV burden (r = 0.366, $p$ = 0.002) (Figure 5D). Finally, PSMA−/EpCAM+ CTCs were significantly associated with worse PFS (HR = 2.10, CI: 1.26–3.49, $p$ = 0.004) and OS (HR = 2.34, CI: 1.24–4.41, $p$ = 0.008), but not MTV burden (r = 0.235, $p$ = 0.054) (Figure 5E).

## Association of EV and CTC subgroups with PSA50 response to $^{177}$Lu-PSMA-617

We next investigated whether EV-derived proteins and CTC subpopulations were associated with treatment sensitivity to $^{177}$Lu-PSMA-617. To do so, we considered treatment response based on PSA50 reductions and used multivariable logistic regression to establish the area under the curve (AUC) when compared with baseline PSA alone or MTV burden alone. We additionally assessed the net reclassification improvement (NRI) to quantify whether inclusion of EV biomarkers improved response classification beyond clinical variables alone. This approach has been applied to advanced prostate cancer, including in the phase 3 Alliance-031201 trial evaluating cell-free tumor DNA alterations, and was found to improve prediction over clinical factors for CRPC patients receiving enzalutamide with or without abiraterone.[30]

In the current study, baseline PSA (AUC: 0.582) and MTV burden (AUC: 0.524) alone showed limited discrimination for PSA50 response. Among individual proteins, the integration of

**Figure 2. Characterization of EVs isolated from patient plasma**

(A) Particle size distribution of isolated EVs measured by nanoparticle tracking analysis (NTA).

(B) Number of proteins detected per patient using data-independent acquisition mass spectrometry (DIA-MS), with a median of 3,767 proteins (range: 2,519–4,555).

(C) Principal-component analysis plot showing technical duplicates for 20 randomly selected samples to monitor assay consistency.

(D) Violin plots showing levels of canonical EV markers across all samples, grouped by functional class: tetraspanins, scaffold markers, lipid raft-associated proteins, and trafficking/secretion regulators.

(E) Subcellular localization of the 5,137 identified EV proteins, revealing their origin from multiple compartments, including cell membrane (18.8%), nuclear (19.5%), cytoplasmic (36.4%), mitochondrial (9.6%), and others (15.7%).

(F) Kaplan-Meier survival analysis showing the prognostic significance of the cumulative detection of targetable surface proteins (PSMA, B7-H3, Trop-2, and STEAP1) stratified into three groups: 0–1 detected proteins, 2 detected proteins, and 3–4 detected proteins. Kaplan-Meier curves were generated using Cox regression analysis for statistical significance ($p$ = 0.0001). The table below provides the number of patients at risk.

(G) The association between increasing number of detectable EV-derived surface proteins (0–1 detected proteins, 2 detected proteins, and 3–4 detected proteins) and molecular tumor volume (MTV) burden. Statistical significance was assessed using the Kruskal-Wallis test. Asterisk marks denote level of statistical significance: **$p$ < 0.01, ***$p$ < 0.001.

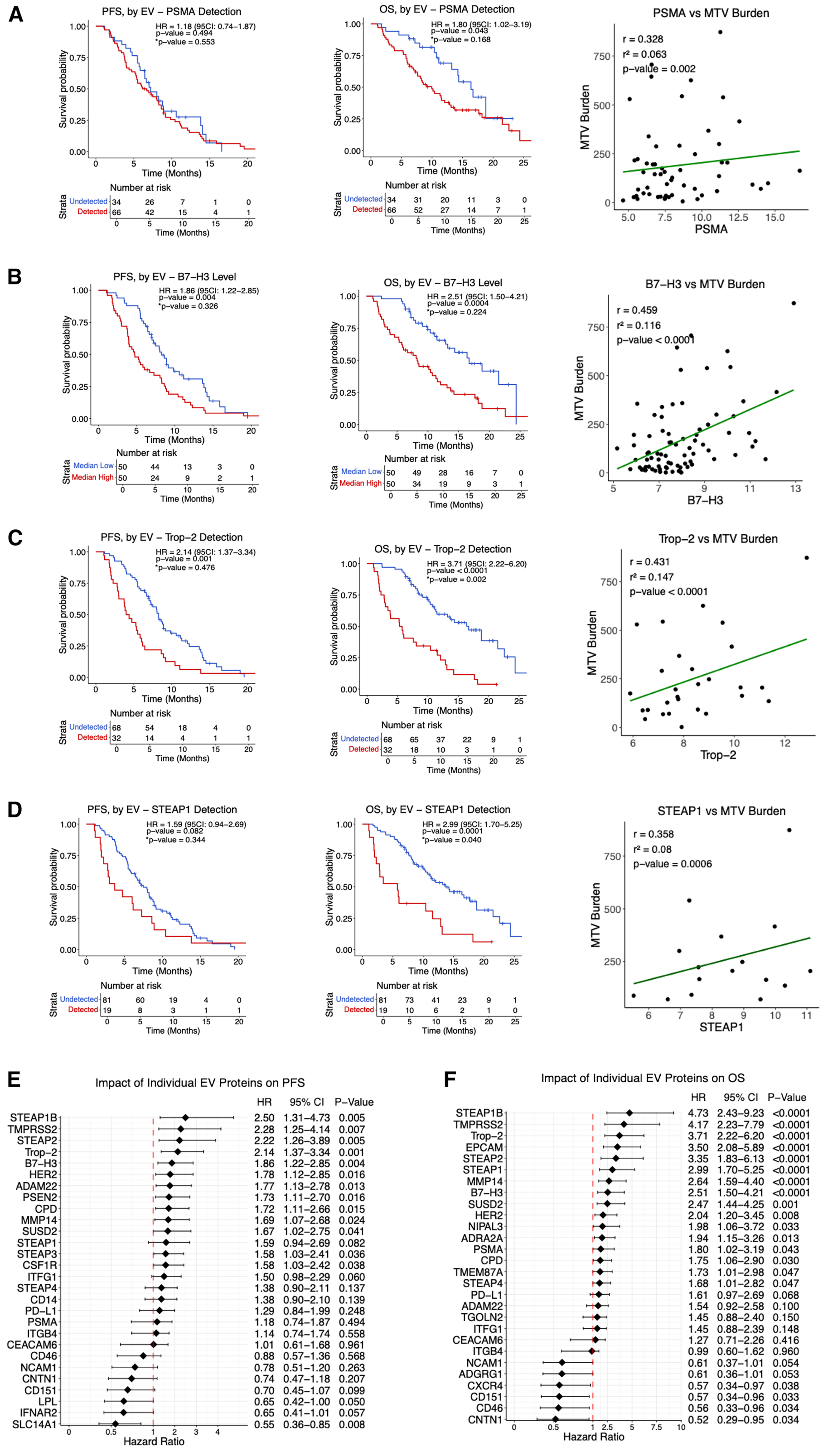


*(legend on next page)*

Trop-2 EVs to baseline PSA (AUC: 0.711, NRI: 56.4%) and MTV burden (AUC: 0.639, NRI: 61%) provided the strongest improvement in PSA50 response prediction. PSMA, B7-H3, and STEAP1 showed modest or no significant improvement to PSA50 response prediction (Table S6).

Beyond canonical tumor markers, an optimized multi-protein EV signature including RAF1, TNKS1BP1, RCCD1, BET1, RPA3, EPB41L5, AHNAK2, PFN2, and MRTFA markedly enhanced prediction when combined with baseline PSA (AUC: 0.878, NRI: 124%), whereas BET1, STARD10, PATJ, RGS3, RPS6KA2, ADAM22, RAF1, LMBRD1, and AHNAK2 EVs significantly enhanced prediction when combined with MTV burden (AUC: 0.893, NRI: 112%) (Table S6).

Among CTC subtypes, only PSMA−/EpCAM+ CTCs (odds ratio = 0.367, CI: 0.139–0.931, $p = 0.038$) showed a significant association with reduced PSA50 response rate. After adjustment, none of the CTC subsets reached statistical significance ($p > 0.05$) (Table S7).

## EV proteins improve prognostic performance beyond baseline PSA levels and MTV burden

We next evaluated whether EV-derived proteins improved prognostic and predictive performance beyond baseline PSA levels based on the calculation of concordance indices (C-indices), in which 0.71–0.80 have been considered very good in modeling clinical and genomic factors derived from other liquid biopsies.[30–33] Baseline PSA alone showed modest prognostic discrimination with C-index of 0.515 for PFS and 0.618 for OS. Incorporating EV proteins into our multivariable model, the addition of B7-H3 EVs and Trop-2 EVs improved the PFS C-index to 0.605 and 0.608 with NRI of 69.6% and 66.1%, respectively. Beyond canonical tumor markers, a multi-protein signature including SRSF1, PCDHGA10, SPINT1, PLK1, SGMS1, ROBO1, TNKS1BP1, EXOSC8, and MAP3K11 (optimized model with 9 proteins) further improved prognostic performance when combined with PSA, achieving a C-index of 0.716 and NRI of 81.1% (Table S8).

For OS prediction, PSA alone demonstrated modest prognostic performance with a C-index of 0.618. The addition of individual EV markers including Trop-2 showed improved C-index discrimination to 0.699 and NRI of 56.1%. The optimized multi-protein signature including SRSF1, SPINT1, Trop-2, MACROH2A2, RPA1, SGMS1, NCAM2, CDCA3, and NDUFB9 when combined with PSA achieved an improved C-index of 0.773 and an NRI of 77.9% (Table S8).

Beyond MTV burden alone (C-index of 0.578 for PFS), the addition of B7-H3 and Trop-2 EVs enhanced the model's performance, raising the C-index to 0.625 and 0.641, with NRI of 71.8% and 63.2%, respectively. The strongest prognostic improvement was achieved with an optimized multi-protein EV signature comprising PLK1, ROBO1, SRSF1, SGMS1, PCDHGA10, SPINT1, EXOSC8, GBP4, and SDCBP2, resulting in a C-index of 0.727 and an NRI of 69.9% (Table S8).

For OS prediction, MTV burden alone showed modest prognostic performance with a C-index of 0.613. The addition of B7-H3 and Trop-2 EVs enhanced the model performance, raising the C-index to 0.674 and 0.695, with NRI of 69.9% and 53.3%, respectively. The strongest improvement was observed with an optimized multi-protein EV signature comprising SRSF1, MACROH2A2, CDCA3, SPINT1, Trop-2, RPA1, PLK1, NCBP1, and SGMS1, achieving a C-index of 0.785 and an NRI of 90.6% (Table S8). These findings highlight the potential of EV-derived protein for refining risk stratification and response prediction, although further external validation is necessary.

## CTC subgroups provide prognostic information beyond PSA and MTV burden

We next assessed if CTCs improved prognostic accuracy beyond routine clinical variables including PSA and MTV burden. For PFS, the addition of total CTC counts (C-index: 0.577 [0.501–0.657]), PSMA+/EpCAM+ (C-index: 0.597 [0.526–0.663]), PSMA+/EpCAM− (C-index: 0.552 [0.469–0.638]), and PSMA−/EpCAM+ (C-index: 0.615 [0.540–0.689]) CTCs all improved the C-index performance beyond PSA levels alone. A similar trend was also observed for OS across all 4 CTC groups.

Furthermore, we investigated the prognostic utility of CTCs beyond MTV burden. Similarly to PSA, the addition of total CTC counts increased the PFS C-index to 0.621 (0.526–0.707) and adding PSMA+/EpCAM+ CTCs increased the C-index to 0.620 (0.529–0.701). The PSMA−/EpCAM+ CTC group again showed the strongest increase in the C-index to 0.624 (0.529–0.713), while the least increase was seen in the PSMA+/EpCAM− CTC group. Similar trends were also seen for OS across the 4 CTC groups. Together, these findings show that CTC subgroups provide prognostic information beyond both PSA and MTV burden (Table S9).

**Figure 3. Prognostic impact and tumor burden associations of EV protein detection and quantification**

(A) Kaplan-Meier curves and scatterplot showing that PSMA detection was significantly associated with worse overall survival (OS), but not progression-free survival (PFS), and was positively correlated with MTV burden (MTV burden, *y* axis; PSMA expression, *x* axis).

(B) Kaplan-Meier curves and scatterplot showing that B7-H3 was associated with shorter PFS and OS and demonstrated a strong positive correlation with MTV burden (MTV burden, *y* axis; B7-H3 expression, *x* axis).

(C) Kaplan-Meier curves and scatterplot of showing that Trop-2 detection was associated with significantly reduced PFS and OS and positively associated with MTV burden (MTV burden, *y* axis; Trop-2 expression, *x* axis).

(D) Kaplan-Meier curves and scatterplot showing that STEAP1 was significantly associated with shorter OS but not PFS and positively correlated with MTV burden (MTV burden, *y* axis; STEAP1 expression, *x* axis).

(E) Forest plot showing proteins that were significantly associated with worse or better PFS. The table to the right indicates the hazard ratio (HR), 95% confidence interval (CI), and *p* value for each protein.

(F) Forest plot showing proteins that were significantly associated with worse or better OS. The table to the right indicates the HR, 95% CI, and *p* value for each protein.

*$p$ value is adjusted for baseline serum PSA level, baseline hemoglobin (Hb), and baseline alkaline phosphatase (ALP).

CellPress
OPEN ACCESS

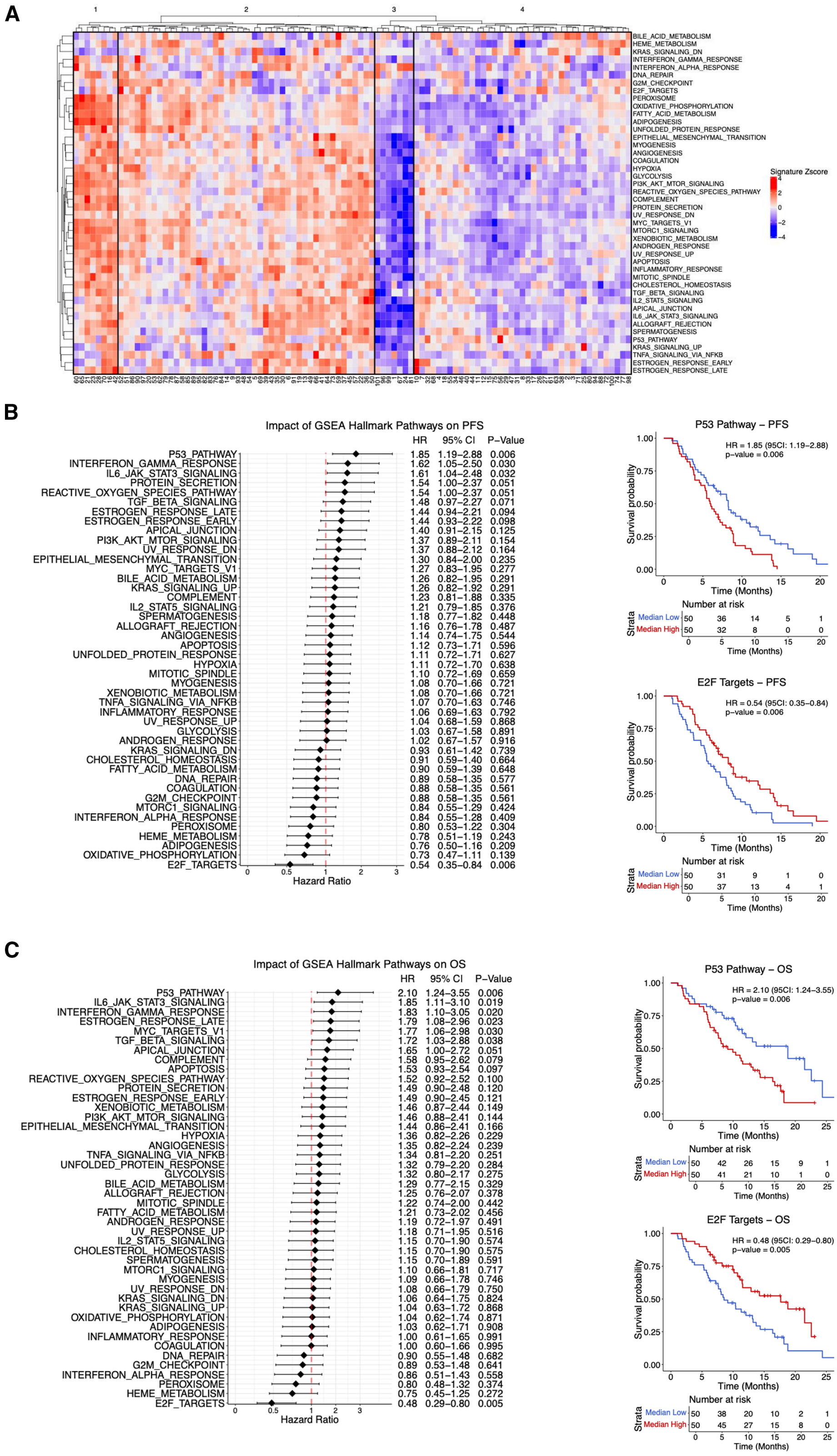


(legend on next page)

## PSMA+ EVs and CTCs provide minimal correlation and limited additive prognostic power

We then evaluated the correlation between PSMA EV expression and all four CTC subgroups. Spearman correlation analysis demonstrated no significant correlation between PSMA EVs and any of the CTC subsets ($p > 0.05$) (Table S10).

We next compared the prognostic performance of PSMA EVs and CTC subsets individually and in combination. For PFS, CTC-based metrics demonstrated stronger prognostic discrimination (C-index range: 0.606–0.625) than PSMA EVs alone (C-index: 0.514 [0.452–0.562]). Combining PSMA EVs with any CTC subset did not improve PFS prediction beyond the CTC models alone. For OS, combining PSMA EVs with total CTC counts increased the C-index to 0.699 (0.607–0.789), and similar improvements were observed when PSMA EVs were combined with individual CTC subsets (Table S11).

## Deconvolution of PSMA- and B7-H3-related protein networks identifies co-expressed proteins as well as divergent biological pathways

To examine global proteomic differences in patients with high EV-derived expression of PSMA, B7-H3, Trop-2, and STEAP1, we conducted a Pearson correlation of these proteins with each other across all samples. This indicated that expression of PSMA, B7-H3, Trop-2, and STEAP1 proteins demonstrated positive correlations with each other (Figure 6A) and with other cell-surface targets such as HER2, but were anti-correlated with the kinesin proteins KIF2A and INPP4B, both of which have been implicated in other cancers.[34,35] Through Spearman correlation, these positive and negative co-expression patterns were also observed across samples (Figure 6B). To conduct deeper analyses through interrogation of protein networks, we adapted the Algorithm for Linking Activity Networks (ALAN) methodology.[36] ALAN effectively cross-compared expression levels of each of these proteins against all other 5,137 proteins within the 100 patient samples in order to uncover strong and weak associations. ALAN similarly indicated that (in reference to PSMA as a benchmark), B7-H3, Trop-2, STEAP1, and HER2 proteins exhibited similar behaviors, whereas KIF2A and INPP4A exhibited inverse correlations with PSMA protein networks (Figure 6C).

To further elucidate the differences between PSMA, B7-H3, Trop-2, and STEAP1 protein networks (separate therapeutic targets in mCRPC drug development), we conducted differential protein expression analysis between the tumors that were PSMA-high/detectable and PSMA-low groups. This indicated that plasma EVs that were PSMA-high/detectable were associated with increased prostate cancer-associated proteins including AMACR (Figure 6D), which has previously been used to diagnose prostate cancer due to its pathognomonic expression in prostatic adenocarcinoma.[37] Similar findings were observed in the Trop-2-detected and B7-H3-high groups. B7-H3-high showed differentially expressed SF3B1, a splicing factor associated with aggressive prostate cancer, and DCPS, a scavenger-decapping enzyme previously implicated in colorectal cancer and glioblastoma biology.[38–40] In contrast, the STEAP1-detectable group demonstrated increased levels of UPP1, a pyrimidine salvage enzyme that has been linked to neutrophil-mediated metastatic niche priming and poor outcomes in preclinical models of metastatic breast cancer.[41] The identification of DCPS and UPP1 and their role in prostate cancer remains largely unexplored. To uncover pathways associated with PSMA and B7-H3, we conducted pre-ranked GSEA on these protein expression profiles based on the 50 Hallmark signaling pathways. This indicated that PSMA was positively associated with reactive oxygen species, while it was negatively associated with mitotic spindle (Figure 5E). B7-H3 was instead significantly associated with suppressed IL2 and IL6 signaling and inflammatory response (Figure 5F). Altogether, our results indicate that EV-derived expression of PSMA and B7-H3 are positively correlated with each other, as well as with Trop-2, STEAP1, and HER2.

## DISCUSSION

Our findings highlight the potential limitations of relying on PSMA-PET imaging metrics alone for prognostication and support the incorporation of plasma proteomic biomarkers to aid in therapeutic decision-making. PSMA-PET parameters such as SUVmean and SUVmax do not reliably predict PFS and OS. While high baseline SUV may predict PSA response, it does not equate to treatment durability, particularly in patients with high metastatic burden. In contrast, PET-derived MTV burden was prognostic, but its quantification is labor intensive and is limited by interpretive variability, particularly in patients with extensive disease.[42] By capturing tumor-derived EV proteins as well as CTC subpopulations concurrently, our assay correlates with serum-based biomarkers such as PSA and alkaline phosphatase, serving as a surrogate for tumor burden and disease biology.

Notably, B7-H3, Trop-2, and STEAP1 were enriched in patients with extensive bone metastases (>20 lesions), potentially making them more suitable targets for more extensive metastatic disease. Additionally, HER2 has surfaced as a significant and

**Figure 4. GSEA pathway-level analysis of plasma EV proteomics reveals associations with clinical outcomes**

(A) Heatmap displaying unsupervised hierarchical clustering of patients based on normalized enrichment scores (NES) from gene set enrichment analysis (GSEA) across Hallmark pathways. Red indicates positive enrichment and blue indicates negative enrichment for each pathway.

(B) (Left) Forest plot showing univariate hazard ratios for individual GSEA pathways associated with progression-free survival (PFS). Hazard ratios (HR), 95% confidence intervals (CI), and $p$ values are shown to the right of each pathway. (Right) Kaplan-Meier survival curves showing that high p53 pathway activity and low E2F target activity (based on median dichotomization of NES) were significantly associated with worse PFS. Survival curves were compared using the log rank test. Patient numbers at risk are indicated below each time point.

(C) (Left) Forest plot showing univariate Cox models for the association between individual GSEA Hallmark pathways and overall survival (OS). HR, 95% CI, and $p$ values are provided for each pathway. (Right) Kaplan-Meier curves demonstrating that high p53 activity and low E2F activity were associated with worse OS. Pathways were dichotomized at the median NES and compared using the log rank test. Number of patients at risk is indicated below each time point.

CellPress
OPEN ACCESS

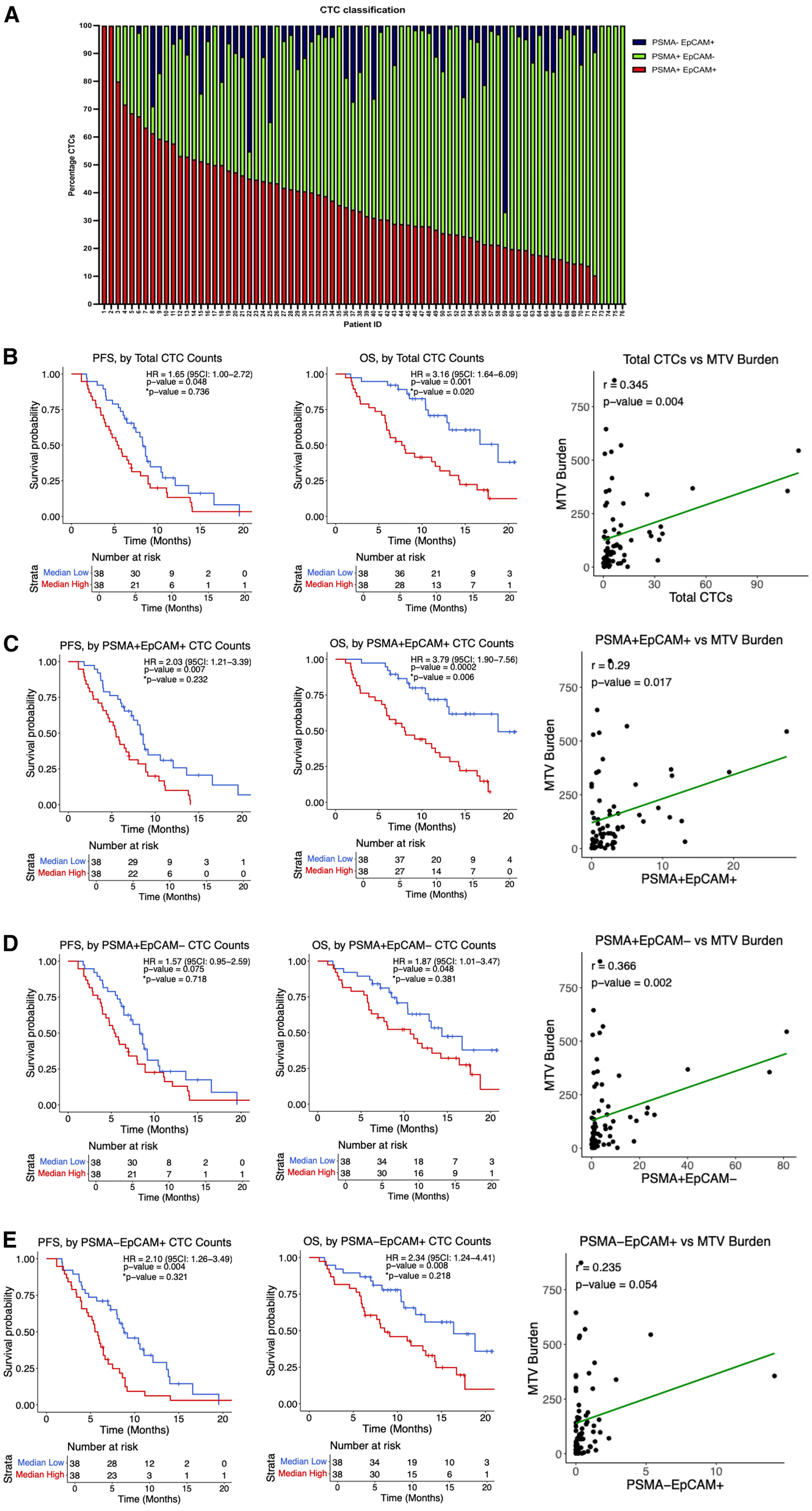


**Figure 5. CTC enumeration and PSMA/EpCAM subtype stratification**

(A) Bar chart displaying the percentage of CTC phenotypes per patient, based on PSMA and EpCAM expression. CTCs were subclassified into PSMA+/EpCAM+ (green), PSMA+/EpCAM− (red), and PSMA−/EpCAM+ (blue).

(B) (Left and middle) Kaplan-Meier survival curves demonstrating that higher total CTC counts were significantly associated with worse PFS and OS. (Right) Scatterplot showing positive correlation between total CTC count (*x* axis) and baseline MTV burden (*y* axis).

(C) (Left and middle) Kaplan-Meier survival curves demonstrating that higher PSMA+ EpCAM+ counts were significantly associated with worse PFS and OS. (Right) Scatterplot showing positive correlation between PSMA+ EpCAM+ (*x* axis) and baseline MTV burden (*y* axis).

(D) (Left and middle) Kaplan-Meier survival curves demonstrating that higher PSMA+ EpCAM− counts were significantly associated with worse OS but not PFS. (Right) Scatterplot showing positive correlation between PSMA+ EpCAM− (*x* axis) and baseline MTV burden (*y* axis).

(E) (Left and middle) Kaplan-Meier survival curves demonstrating that higher PSMA− EpCAM+ counts were significantly associated with worse PFS and OS. (Right) Scatterplot showing positive correlation between PSMA− EpCAM+ (*x* axis) and baseline MTV burden (*y* axis).

**p* value is adjusted for baseline serum PSA level, baseline hemoglobin (Hb), and baseline alkaline phosphatase (ALP).

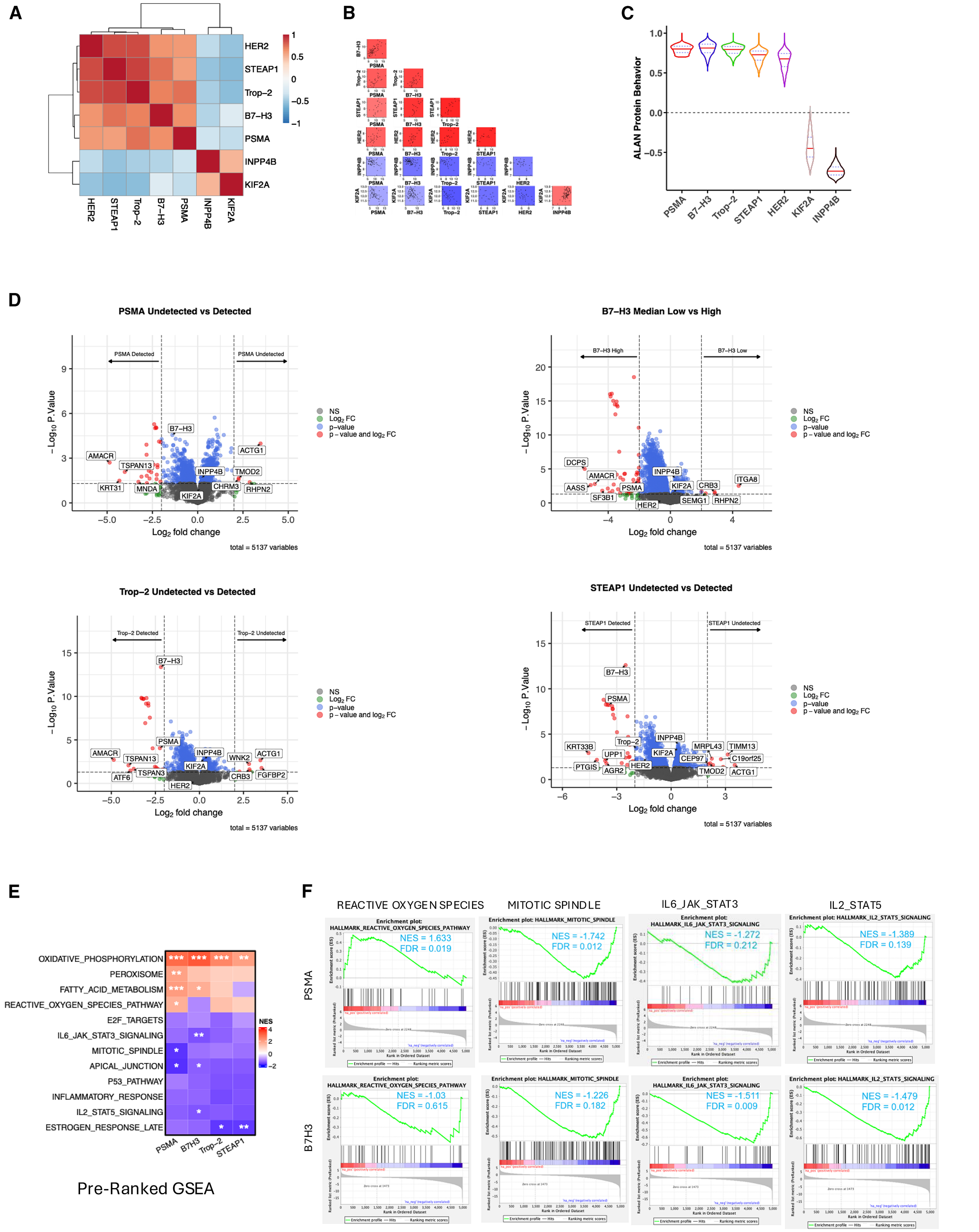


(legend on next page)

CellPress
OPEN ACCESS

clinically relevant protein marker in our analysis. Its elevated expression is notably linked to poorer OS. This aligns with existing research suggesting a role for HER2 in prostate cancer progression and resistance, opening the door to HER2-targeted strategies in mCRPC.[43]

Intriguingly, our results suggest that elevated canonical p53 signaling may confer resistance to therapies inducing single-stranded DNA breaks (SSBs) via beta-particle emission, which are efficiently mended by base excision repair (BER) rather than triggering apoptosis.[44,45] Activated by ATM/ATR kinase signaling, wild-type p53 upregulates genes such as p21, promoting cell-cycle arrest and enhancing DNA repair functions.[46] By increasing BER, wild-type p53 may enable tumor cells to recover from sublethal SSBs, reducing the therapeutic effectiveness of $^{177}$Lu-PSMA-617. On the other hand, impaired canonical p53 pathway function may lead to more cancer cell death due to the inability to repair SSBs induced by beta-particle radiotherapeutics, manifesting clinically as enhanced sensitivity to $^{177}$Lu-PSMA-617.

Conversely, high E2F pathway activity was associated with improved outcomes in our study, suggesting a potential link between tumor proliferation and increased sensitivity to $^{177}$Lu-PSMA-617. The E2F transcriptional program, normally kept in check by RB1, promotes cell-cycle progression and entry into S-phase and can drive replication stress when dysregulated.[47] Although RB1 genomic status was not assessed in our cohort, functional loss of RB1 is known to impair DNA double-strand break repair and disrupt cell-cycle checkpoints, leading to the accumulation of unrepaired DNA damage following radiation. Radiation is most effective against actively dividing cells, particularly those in G2/M, where limited repair time before mitosis increases the likelihood of lethal damage, whereas quiescent or slowly cycling cells tend to be more resistant.[48,49] Elevated E2F activity accelerates S-phase entry, raising the probability that radiation-induced single-strand breaks (SSBs) will occur during DNA synthesis, potentially exceeding the capacity of BER and resulting in mitotic catastrophe.[50] Overall, these findings suggest that elevated E2F signaling may represent a biologically meaningful state of tumor vulnerability to PSMA-targeted radiotherapy, rather than a feature of treatment resistance, helping to explain the improved outcomes seen in this subgroup. This finding is in contrast to other systemic therapies used in mCRPC, including hormonal or chemotherapeutic agents, wherein activation of the E2F pathway has been associated with inferior outcomes.[51]

The analysis of CTC subpopulations revealed that the presence of high PSMA+/EpCAM+ CTCs counts was associated with worse PFS and OS, consistent with prior studies linking higher PSMA+ CTC counts with unfavorable outcomes.[11] An observation in the context of PSMA-targeted therapies was the detection of PSMA−/EpCAM+ CTCs, which was also associated with worse PFS and OS, suggesting that these cells represent dedifferentiated phenotypes such as androgen receptor (AR)-negative neuroendocrine (NE)-negative (AR− NE−) or AR-negative neuroendocrine-positive (AR− NE+) prostate cancer. These findings underscore the presence of PSMA− CTCs alongside PSMA+ CTCs within the same individuals, highlighting intra-patient heterogeneity, a critical barrier to effective systemic therapy. This further supports the notion that dual-targeting strategies, such as those combining PSMA- and B7-H3-directed therapies, may be needed to address disease heterogeneity.

In summary, we provide prospective evidence that blood-based EV proteomic analysis can uncover clinically relevant biomarkers and signaling pathways potentially associated with treatment response and resistance to $^{177}$Lu-PSMA-617 radioligand therapy. Our findings lay the foundation for future trials incorporating EV-based proteomics into clinical workflows, ultimately enabling more precise biology-driven patient selection for individuals with advanced prostate cancer.

## Limitations of the study

Our study has several limitations. It was conducted at a single institution, which may limit the generalizability or reproducibility of our findings. The study cohort comprised 92% white patients, which constrains the applicability of these findings to other population groups. The absence of a validation cohort highlights the need for future confirmatory studies, which are currently being planned by our group and others. Importantly, the modeled protein signatures associated with PSA50 response, PFS, and OS require independent validation in external cohorts to establish their predictive and prognostic utility. The optimized multi-protein EV signatures identified through LASSO-based feature selection are exploratory, derived from a single cohort, and vary in composition across endpoints (PSA50, PFS, and OS) and clinical backbones (PSA and MTV burden). Proteome-wide feature selection is inherently prone to identifying endpoint-specific associations in a single cohort, raising uncertainty about the biological stability and reproducibility of these panels in independent cohorts. Although internal bootstrap optimism correction was applied, external validation in independent mCRPC cohorts receiving $^{177}$Lu-PSMA-617 is required to confirm the stability and clinical utility of these signatures. Because the proteomic analyses are descriptive rather than confirmatory, and because the associations with PFS and OS were sometimes consistent but not uniform across markers, these findings should be interpreted cautiously. In addition, our EV isolation protocol does not differentiate

**Figure 6. Deconvolution of EV protein networks reveals divergent biology between PSMA and B7-H3**

(A and B) Correlation matrices using Pearson correlation show positive co-expression of PSMA, B7-H3, Trop-2, STEAP1, and HER2, and negative correlations with KIF2A and INPP4B across patient samples.

(C) Violin plots showing ALAN behavior scores for EV surface proteins of interest, with similar network behavior among PSMA, B7-H3, Trop-2, STEAP1, and HER2 and inverse relationships for KIF2A and INPP4B.

(D) Volcano plots from differential protein expression analysis between PSMA-detected vs. PSMA-undetected EV samples (left) and B7-H3-high vs. B7-H3-low EV samples (right). Both PSMA and B7H3-high EVs showed elevated AMACR, while B7-H3-high EVs were enriched for SF3B1 and DCPS.

(E) Heatmap summarizing Hallmark pathway enrichment by pre-ranked GSEA comparing PSMA and B7-H3-high EV protein profiles. Asterisk marks denote level of statistical significance: *$q < 0.05$, **$q < 0.01$, ***$q < 0.001$.

(F) GSEA enrichment plots reveal the upregulated and downregulated pathways in PSMA and B7-H3-high EV samples.

tumor-derived EVs from non-tumor derived vesicles. Ultracentrifugation isolated heterogeneous vesicle populations, can damage vesicles, promote aggregation, and produce variable recovery and yield across samples, and these shortcomings should be considered when interpreting EV-associated signals. While technical replicates confirm instrument-level reproducibility, pre-analytical variability inherent to ultracentrifugation-based EV isolation, including sample-dependent differences in yield and purity, was not fully accounted for. Independent workflow replicates processed from plasma through to mass spectrometry acquisition are critical for establishing robust biomarker discovery pipelines and represent a limitation of this study. Although label-free quantification (LFQ) intensities were normalized using NTA-derived total EV particle counts, NTA measurements following ultracentrifugation may include co-isolated non-EV particles such as lipoproteins or protein aggregates. While this approach avoids the compositional confounding associated with protein abundance-based normalization, co-isolation of non-EV particles represents a known limitation of ultracentrifugation-based EV isolation and may introduce variability in the normalization factor across samples. Several key EV proteins were modeled using binary detection rather than continuous abundance, which may introduce ambiguity near the limit of detection. The use of detected/undetected and median-based dichotomization for EV protein classification is appropriate for exploratory discovery in a single cohort and reduces noise near the detection limit, but these binary thresholds are inherently assay, workflow, and cohort-specific, limiting their direct translational applicability. Without independent workflow replicates and analytically locked detection thresholds, these binary features may partly reflect pre-analytical variability and tumor burden rather than reproducible biological signals, and continuous abundance-based modeling with prospectively defined thresholds are necessary to advance these findings toward clinical utility. *Detection status* may reflect variability in EV recovery, mass spectrometry sensitivity, protein abundance near the detection threshold, or true biological absence. At present, no established benchmarks define analytical sensitivity for EV protein detection in plasma from patients with mCRPC, representing a broader technical limitation in the field. Improving assay sensitivity and dynamic range remains a key objective toward the development of a clinical-grade EV proteomics assay. Furthermore, some EV proteins were detected in only a minority of patients, and, although we used a 10% detection threshold for inclusion given the exploratory nature of this study, the prognostic value of proteins with low detection prevalence remains limited. It also remains unknown whether low detection reflects true tumor biology absence within EVs or insufficient sensitivity of the mass spectrometry workflow, and this uncertainty may influence the interpretation of prognostic associations. The observation that approximately 1,600 proteins are unique to cancer samples relative to the 20 healthy donor controls likely reflects a combination of factors including limited control sample size, differences in proteome depth, and missingness patterns rather than strictly tumor-derived EV specificity, and this proportion is difficult to reconcile with the expected minority fraction of tumor-derived EV material in plasma following ultracentrifugation-based enrichment. While several of these cancer-enriched proteins, including PSMA, B7-H3, and STEAP1, are detected in prostate cancer cell lines supporting their tumor relevance, the broader functional and biological significance of the full set of 1,600 cancer-enriched proteins has not been fully characterized and remains an area of active investigation by our group and others.

While DIA-MS enables deep proteomic coverage and pathway analysis, its use in clinical practice remains limited. Future efforts could focus on developing EV proteomic assays using platforms such as Olink or ELISA, which offer greater scalability and are more compatible with a rapid clinical workflow. In terms of the GSEA pathway analyses, we acknowledge that these Hallmark profiles were originally constructed based on transcriptomic (not proteomic) data; therefore, key differential pathways require further validation. Furthermore, our cohort included only patients with PSMA-predominant disease, which limited the detection of rare neuroendocrine markers such as DLL3. Conversely, predominantly nuclear-localized proteins such as AR, FOXA1, and HOXB13 are under-represented in EVs and their lower abundance in EVs does not reflect their concentration in tumor cells. Additionally, without a validation cohort, the pathway-level interpretations and the prognostic associations linking EV proteins to PFS and OS analysis require further confirmation. Finally, because liquid biopsy biomarkers frequently track with disease burden, disentangling disease burden effects from true biological variation is inherently challenging in EV studies, and this complexity should be considered when interpreting our results. This work requires prospective validation in other mCRPC patient cohorts, preferably those involving $^{177}$Lu-PSMA-617 or other PSMA-targeting therapeutics. In particular, the provocative finding of potential increased sensitivity to $^{177}$Lu-PSMA-617 when the E2F pathway is upregulated, or potential decreased sensitivity when the p53 pathway is upregulated, requires independent confirmation.

## RESOURCE AVAILABILITY

### Lead contact

Further information and requests should be directed to and will be fulfilled by the lead contact, Dr. Emmanuel Antonarakis (anton401@umn.edu).

### Materials availability

No plasmids, mouse lines, or other biological reagents were generated in this study. The EV isolation protocol using differential ultracentrifugation described in this study is available in previously published work (DOI: https://doi.org/10.3390/cancers16244261).

### Data and code availability

- Proteomic.Raw files derived from plasma EV samples have been deposited at the ProteomeXchange Consortium via PRIDE.
- Raw files can be downloaded using an FTP client using this link: https://massive.ucsd.edu/ProteoSAFe/dataset.jsp?task=0e236e1d0c15483a94c996ec937bd0b8. EV protein intensity and CTC enumeration data for each patient were included as a supplemental table.
- This paper does not report original code.
- Any additional information required to reanalyze the data reported in this paper is available from the lead contact upon request.

## ACKNOWLEDGMENTS

This study was partially funded by an investigator-initiated study grant (to E.S.A.) sponsored by Novartis (CAAA617A1US11T). A.T.A. is supported by a

CellPress
OPEN ACCESS

Postdoctoral Fellowship, PF-23-1153194-01-CDP, grant #: (https://doi.org/10.53354/ACS.PF-23-1153194-01-CDP.pc.gr.175399). N.A.Z. was supported by a 2022 Department of Defense Early Investigator Award (W81XWH-22-1-0242), a 2025 Department of Defense Physician Research Award (HT9425-25-1-0438), a 2022 Prostate Cancer Foundation Young Investigator Award, the University of Minnesota Institute for Prostate and Urologic Cancers Philanthropic Fund, and The Randy Shaver Community Cancer Fund (2023, 2024, and 2025). Support for P.W.V. was provided by R50CA211256. S.M.D. is supported by NIH grants R01CA174777 and R01CA270539. J.H.H. is supported by the American Cancer Society (IRG-21-049-61-IRG) and NCI/NIH (R37 1R37CA288972-01). J.M.D. is supported by the Masonic Cancer Center and The Department of Pharmacology Basic/Clinical Pilot Research Award (BCPRA) at the University of Minnesota and by the NIH/NCI STTR R41CA268344-01. E.S.A. is supported by NCI grant P30 CA077598 and Department of Defense grant W81XWH-22-2-0025. Mass spectrometry data were acquired in the Analytical Biochemistry Shared Resource of the Masonic Cancer Center, which is funded in part by the Cancer Center Support Grant P30CA077598. Part of statistical analyses was carried out in the Biostatistics Shared Resource of the Masonic Cancer Center, supported in part by the NCI Cancer Center Support grant P30CA077598 and the National Center for Advancing Translational Sciences of the NIH award number UM1TR004405. The content is solely the responsibility of the authors and does not necessarily represent the official views of the National Institutes of Health. Portions of this work were conducted in the Minnesota Nano Center, which is supported by the National Science Foundation through the National Nanotechnology Coordinated Infrastructure (NNCI) under award number ECCS-2025124. The figure schematic was created with https://biorender.com.

## AUTHOR CONTRIBUTIONS

Conceptualization, A.T.A., M.L.L., S.M.D., J.H.H., J.M.D., and E.S.A.; methodology, E.B., L.K., J.H.H., J.M.D., and E.S.A.; investigation, A.T.A., S.B., K.M.S., T.Y., G.J., I.O., C.J.R., N.A.Z., D.S., S.M.D., J.H.H., J.M.D., and E.S.A.; writing – original draft, A.T.A., E.B., L.K., Y.Z., P.W.V., and J.H.H.; writing – review & editing, Y.Z., Z.C., D.S., C.J.R., N.A.Z., S.M.D., J.H.H., J.M.D., and E.S.A.; funding acquisition, A.T.A., S.M.D., J.H.H., J.M.D., and E.S.A.; resources, Y.Z., P.W.V., Z.C., J.H.H., J.M.D., and E.S.A.; supervision, Z.C., S.M.D., J.H.H., J.M.D., and E.S.A.

## DECLARATION OF INTERESTS

E.B. serves as a consultant for Astrin Biosciences, Tempus AI, and EMRGNSE. J.H. consults for Tempus AI and Astrin Biosciences and is a co-founder of EMRGNSE LLC. J.M.D. serves as Chief Scientific Officer of Astrin Biosciences with stock options. The interest related to J.M.D. has been reviewed and managed by the University of Minnesota in accordance with its conflict-of-interest policies. N.A.Z. has received advisory fees from Bayer (Institutional) and Johnson and Johnson (Institutional) and consulting fees from Slingshot Insights and Mosaic. E.S.A. reports grants and personal fees from Janssen, Johnson & Johnson, Sanofi, Bayer, Bristol Myers Squibb, Convergent Therapeutics, Curium, MacroGenics, Merck, Pfizer, and AstraZeneca; personal fees from Aadi Bioscience, Abeona Therapeutics, Aikido Pharma, Astellas, Amgen, Blue Earth, Boundless Bio, Corcept Therapeutics, Duality Bio, Exact Sciences, Hookipa Pharma, Invitae, Eli Lilly, Foundation Medicine, Menarini-Silicon Biosystems, Tango Therapeutics, Tempus, Tolmar Scientific, VIR Biotechnology, and Z-alpha; and grants from Novartis, Celgene, and Orion. E.S.A. also has a patent for an AR-V7 biomarker technology that has been licensed to QIAGEN.

## STAR★METHODS

Detailed methods are provided in the online version of this paper and include the following:

- KEY RESOURCES TABLE
- EXPERIMENTAL MODEL AND STUDY PARTICIPANT DETAILS
  - Study participants
  - Cancer free controls
- METHOD DETAILS
  - EV isolation
  - Mass spectrometry
  - Processing of mass spectrometry proteomic data
  - Nanotracker analysis
  - Protein compartmentalization
  - CTC enumeration
  - Clinical outcomes
  - PSMA-PET imaging
- QUANTIFICATION AND STATISTICAL ANALYSIS
  - Biostatistics analysis
  - Prognostic modeling and internal validation
  - Differential protein expression
  - GSEA
  - ALAN

## SUPPLEMENTAL INFORMATION

Supplemental information can be found online at https://doi.org/10.1016/j.xcrm.2026.102764.

Received: August 15, 2025
Revised: November 27, 2025
Accepted: March 24, 2026
Published: April 20, 2026

## REFERENCES

1. Mateo, J., and Zurita, A.J. (2024). 177Lu-PSMA-617 for metastatic prostate cancer: aiming for the right spot. Lancet Lond. Engl. *404*, 1174–1176.
2. Kratochwil, C., Giesel, F.L., Stefanova, M., Benešová, M., Bronzel, M., Afshar-Oromieh, A., Mier, W., Eder, M., Kopka, K., and Haberkorn, U. (2016). PSMA-Targeted Radionuclide Therapy of Metastatic Castration-Resistant Prostate Cancer with 177Lu-Labeled PSMA-617. J. Nucl. Med. *57*, 1170–1176.
3. Sartor, O., de Bono, J., Chi, K.N., Fizazi, K., Herrmann, K., Rahbar, K., Tagawa, S.T., Nordquist, L.T., Vaishampayan, N., El-Haddad, G., et al. (2021). Lutetium-177-PSMA-617 for Metastatic Castration-Resistant Prostate Cancer. N. Engl. J. Med. *385*, 1091–1103.
4. Morris, M.J., Castellano, D., Herrmann, K., de Bono, J.S., Shore, N.D., Chi, K.N., Crosby, M., Piulats, J.M., Fléchon, A., Wei, X.X., et al. (2024). 177Lu-PSMA-617 versus a change of androgen receptor pathway inhibitor therapy for taxane-naive patients with progressive metastatic castration-resistant prostate cancer (PSMAfore): a phase 3, randomised, controlled trial. Lancet Lond. Engl. *404*, 1227–1239.
5. Novartis Pluvicto™ Demonstrates Statistically Significant and Clinically Meaningful rPFS Benefit in Patients with PSMA-Positive Metastatic Hormone-Sensitive Prostate Cancer. (2025).
6. Murthy, V., Voter, A.F., Nguyen, K., Allen-Auerbach, M., Chen, L., Caputo, S., Ledet, E., Akerele, A., Moradi Tuchayi, A., Lawhn-Heath, C., et al. (2024). Efficacy and Toxicity of [177Lu]Lu-PSMA-617 for Metastatic Castration-Resistant Prostate Cancer: Results from the U.S. Expanded-Access Program and Comparisons with Phase 3 VISION Data. J. Nucl. Med. *65*, 1740–1744.
7. Emmett, L., Papa, N., Subramaniam, S., Crumbaker, M., Joshua, A.M., Nguyen, A., Weickhardt, A.J., Lee, S.T., Ng, S., Francis, R.J., et al. (2025). Predictive and prognostic value of baseline PSMA-PET total tumor volume and SUV mean within ENZA-p, a randomized phase II trial of enzalutamide versus enzalutamide plus [ $^{177}$ Lu] Lu-PSMA-617 (ANZUP1901). J. Clin. Oncol. *43*, 5011.
8. Giunta, E.F., Marini, I., Sarnelli, A., Brighi, N., Foca, F., Schepisi, G., Lolli, C., Monti, M., Nicolini, S., Cursano, M.C., et al. (2025). Serum biomarkers in metastatic castration-resistant prostate cancer (mCRPC) patients receiving [ $^{177}$ Lu]Lu-PSMA-617 therapy: Post hoc analysis of a phase II clinical trial. J. Clin. Oncol. *43*, 190.

9. Gafita, A., Voter, A., Shesadri, S., Spitz, A., Marshall, C.H., Rowe, S.P., Markowski, M.C., Pomper, M.G., Civelek, A.C., Carducci, M.A., et al. (2024). Initial Experience with [177Lu]Lu-PSMA-617 After Regulatory Approval for Metastatic Castration-Resistant Prostate Cancer: Efficacy, Safety, and Outcome Prediction. J. Nucl. Med. *65*, 1724–1730.

10. Deek, M.P., Van der Eecken, K., Sutera, P., Deek, R.A., Fonteyne, V., Mendes, A.A., Decaestecker, K., Kiess, A.P., Lumen, N., Phillips, R., et al. (2022). Long-Term Outcomes and Genetic Predictors of Response to Metastasis-Directed Therapy Versus Observation in Oligometastatic Prostate Cancer: Analysis of STOMP and ORIOLE Trials. J. Clin. Oncol. *40*, 3377–3382.

11. Tagawa, S.T., Thomas, C., Sartor, A.O., Sun, M., Stangl-Kremser, J., Bissassar, M., Vallabhajosula, S., Huicochea Castellanos, S., Nauseef, J.T., Sternberg, C.N., et al. (2024). Prostate-Specific Membrane Antigen-Targeting Alpha Emitter via Antibody Delivery for Metastatic Castration-Resistant Prostate Cancer: A Phase I Dose-Escalation Study of 225Ac-J591. J. Clin. Oncol. *42*, 842–851.

12. Shenderov, E., De Marzo, A.M., Lotan, T.L., Wang, H., Chan, S., Lim, S.J., Ji, H., Allaf, M.E., Chapman, C., Moore, P.A., et al. (2023). Neoadjuvant enoblituzumab in localized prostate cancer: a single-arm, phase 2 trial. Nat. Med. *29*, 888–897.

13. Bardia, A., Messersmith, W.A., Kio, E.A., Berlin, J.D., Vahdat, L., Masters, G.A., Moroose, R., Santin, A.D., Kalinsky, K., Picozzi, V., et al. (2021). Sacituzumab govitecan, a Trop-2-directed antibody-drug conjugate, for patients with epithelial cancer: final safety and efficacy results from the phase I/II IMMU-132-01 basket trial. Ann. Oncol. *32*, 746–756.

14. Kelly, W.K., Danila, D.C., Lin, C.C., Lee, J.L., Matsubara, N., Ward, P.J., Armstrong, A.J., Pook, D., Kim, M., Dorff, T.B., et al. (2024). Xaluritamig, a STEAP1 × CD3 XmAb 2+1 Immune Therapy for Metastatic Castration-Resistant Prostate Cancer: Results from Dose Exploration in a First-in-Human Study. Cancer Discov. *14*, 76–89.

15. Veerman, R.E., Teeuwen, L., Czarnewski, P., Güclüler Akpinar, G., Sandberg, A., Cao, X., Pernemalm, M., Orre, L.M., Gabrielsson, S., and Eldh, M. (2021). Molecular evaluation of five different isolation methods for extracellular vesicles reveals different clinical applicability and subcellular origin. J. Extracell. Vesicles *10*, e12128.

16. Wang, X., Tian, L., Lu, J., and Ng, I.O.-L. (2022). Exosomes and cancer - Diagnostic and prognostic biomarkers and therapeutic vehicle. Oncogenesis *11*, 54.

17. Yáñez-Mó, M., Siljander, P.R.M., Andreu, Z., Zavec, A.B., Borràs, F.E., Buzas, E.I., Buzas, K., Casal, E., Cappello, F., Carvalho, J., et al. (2015). Biological properties of extracellular vesicles and their physiological functions. J. Extracell. Vesicles *4*, 27066.

18. Casanova-Salas, I., Aguilar, D., Cordoba-Terreros, S., Agundez, L., Brandariz, J., Herranz, N., Mas, A., Gonzalez, M., Morales-Barrera, R., Sierra, A., et al. (2024). Circulating tumor extracellular vesicles to monitor metastatic prostate cancer genomics and transcriptomic evolution. Cancer Cell *42*, 1301–1312.e7.

19. Arafa, A.T., Ludwig, M., Tuncer, O., Kollitz, L., Gustafson, A., Boytim, E., Luo, C., Sabal, B., Steinberger, D., Zhao, Y., et al. (2024). Isolation of Plasma Extracellular Vesicles for High-Depth Analysis of Proteomic Biomarkers in Metastatic Castration-Resistant Prostate Cancer Patients. Cancers *16*, 4261.

20. Andrews, J.R., Kim, Y., Horjeti, E., Arafa, A., Gunn, H., De Bruycker, A., Phillips, R., Song, D., Childs, D.S., Sartor, O.A., et al. (2025). PSMA+ Extracellular Vesicles Are a Biomarker for SABR in Oligorecurrent Prostate Cancer: Analysis from the STOMP-like and ORIOLE Trial Cohorts. Clin. Cancer Res. *31*, 1142–1149.

21. Li, J., Smith, L.S., and Zhu, H.-J. (2021). Data-independent acquisition (DIA): An emerging proteomics technology for analysis of drug-metabolizing enzymes and transporters. Drug Discov. Today Technol. *39*, 49–56.

22. Kitata, R.B., Yang, J.-C., and Chen, Y.-J. (2023). Advances in data-independent acquisition mass spectrometry towards comprehensive digital proteome landscape. Mass Spectrom. Rev. *42*, 2324–2348.

23. Sharifi, M.N., Sperger, J.M., Taylor, A.K., Tippins, K.E., Reese, S.R., Carreno, V., Kaufmann, K.R., Chang, A.H., Nunamaker, L.A., Linebarger, C., et al. (2025). High-Purity CTC RNA Sequencing Identifies Prostate Cancer Lineage Phenotypes Prognostic for Clinical Outcomes. Cancer Discov. *15*, 969–987. https://doi.org/10.1158/2159-8290.CD-24-1509.

24. Gupta, S., Halabi, S., Yang, Q., Roy, A., Tubbs, A., Gore, Y., George, D.J., Nanus, D.M., Antonarakis, E.S., Danila, D.C., et al. (2023). PSMA-positive Circulating Tumor Cell Detection and Outcomes with Abiraterone or Enzalutamide Treatment in Men with Metastatic Castrate-resistant Prostate Cancer. Clin. Cancer Res. *29*, 1929–1937.

25. Armstrong, A.J., Halabi, S., Luo, J., Nanus, D.M., Giannakakou, P., Szmulewitz, R.Z., Danila, D.C., Healy, P., Anand, M., Rothwell, C.J., et al. (2019). Prospective Multicenter Validation of Androgen Receptor Splice Variant 7 and Hormone Therapy Resistance in High-Risk Castration-Resistant Prostate Cancer: The PROPHECY Study. J. Clin. Oncol. *37*, 1120–1129.

26. Antonarakis, E.S., Lu, C., Wang, H., Luber, B., Nakazawa, M., Roeser, J. C., Chen, Y., Mohammad, T.A., Chen, Y., Fedor, H.L., et al. (2014). AR-V7 and resistance to enzalutamide and abiraterone in prostate cancer. N. Engl. J. Med. *371*, 1028–1038.

27. Mallery, K., Bristow, N.R., Heller, N., Travadi, Y., Arafa, A., Kamalanathan, K., Galeano-Garces, C., Ahmadi, M., Schaap, G., Hesch, A., et al. (2025). Circulating tumor cell detection in cancer patients using in-flow deep learning holography. arXiv, . Preprint at. https://doi.org/10.48550/arXiv.2507.06536.

28. Buteau, J.P., Martin, A.J., Emmett, L., Iravani, A., Sandhu, S., Joshua, A. M., Francis, R.J., Zhang, A.Y., Scott, A.M., Lee, S.T., et al. (2022). PSMA and FDG-PET as predictive and prognostic biomarkers in patients given [177Lu]Lu-PSMA-617 versus cabazitaxel for metastatic castration-resistant prostate cancer (TheraP): a biomarker analysis from a randomised, open-label, phase 2 trial. Lancet Oncol. *23*, 1389–1397.

29. Duriseti, S., Berenji, G., Tsai, S., Rettig, M., and Nickols, N.G. (2023). Quantitative assessment of PSMA PET response to therapy in castration-sensitive prostate cancer using an automated imaging platform for disease identification and measurement. Eur. J. Hybrid Imaging *7*, 7.

30. Halabi, S., Guo, S., Luo, B., Yu, C., Knutson, T.P., Kobilka, A., Lyman, J., Beltran, H., Antonarakis, E.S., Galsky, M.D., et al. (2026). Pathogenic Genomic Alterations in Circulating Tumor DNA Predict Overall Survival in Men with Metastatic Castrate-resistant Prostate Cancer. Eur. Urol. *89*, 332–342. https://doi.org/10.1016/j.eururo.2025.07.011.

31. Halabi, S., Lin, C.Y., Kelly, W.K., Fizazi, K.S., Moul, J.W., Kaplan, E.B., Morris, M.J., and Small, E.J. (2014). Updated prognostic model for predicting overall survival in first-line chemotherapy for patients with metastatic castration-resistant prostate cancer. J. Clin. Oncol. *32*, 671–677.

32. Chi, K.N., Kheoh, T., Ryan, C.J., Molina, A., Bellmunt, J., Vogelzang, N.J., Rathkopf, D.E., Fizazi, K., Kantoff, P.W., Li, J., et al. (2016). A prognostic index model for predicting overall survival in patients with metastatic castration-resistant prostate cancer treated with abiraterone acetate after docetaxel. Ann. Oncol. *27*, 454–460.

33. Gafita, A., Calais, J., Grogan, T.R., Hadaschik, B., Wang, H., Weber, M., Sandhu, S., Kratochwil, C., Esfandiari, R., Tauber, R., et al. (2021). Nomograms to predict outcomes after 177Lu-PSMA therapy in men with metastatic castration-resistant prostate cancer: an international, multicentre, retrospective study. Lancet Oncol. *22*, 1115–1125.

34. Wang, J., Ma, S., Ma, R., Qu, X., Liu, W., Lv, C., Zhao, S., and Gong, Y. (2014). KIF2A silencing inhibits the proliferation and migration of breast cancer cells and correlates with unfavorable prognosis in breast cancer. BMC Cancer *14*, 461.

35. Rodgers, S.J., Ooms, L.M., Oorschot, V.M.J., Schittenhelm, R.B., Nguyen, E.V., Hamila, S.A., Rynkiewicz, N., Gurung, R., Eramo, M.J., Sriratana, A., et al. (2021). INPP4B promotes PI3Kα-dependent late endosome formation and Wnt/β-catenin signaling in breast cancer. Nat. Commun. *12*, 3140.

36. Bergom, H.E., Shabaneh, A., Day, A., Ali, A., Boytim, E., Tape, S., Lozada, J.R., Shi, X., Kerkvliet, C.P., McSweeney, S., et al. (2023). ALAN is a

computational approach that interprets genomic findings in the context of tumor ecosystems. Commun. Biol. *6*, 417.

37. Jiang, N., Zhu, S., Chen, J., Niu, Y., and Zhou, L. (2013). A-methylacyl-CoA racemase (AMACR) and prostate-cancer risk: a meta-analysis of 4,385 participants. PLoS One *8*, e74386.

38. Jiménez-Vacas, J.M., Herrero-Aguayo, V., Gómez-Gómez, E., León-González, A.J., Sáez-Martínez, P., Alors-Pérez, E., Fuentes-Fayos, A.C., Martínez-López, A., Sánchez-Sánchez, R., González-Serrano, T., et al. (2019). Spliceosome component SF3B1 as novel prognostic biomarker and therapeutic target for prostate cancer. Transl. Res. J. Lab. Clin. Med. *212*, 89–103.

39. Duan, H., Xie, Y., Wu, S., Zhao, G., Zeng, Z., Hu, H., Yu, Y., Hu, W., Yang, Y., Chen, Y., et al. (2024). Effect of the mRNA decapping enzyme scavenger (DCPS) inhibitor RG3039 on glioblastoma. J. Transl. Med. *22*, 880.

40. Jan Dimberg, null; Shamoun, L., Johansson, G., Landerholm, K., and Wågsäter, D. (2024). Emerging role and clinical implication of mRNA scavenger decapping enzyme in colorectal cancer. Pathol. Res. Pract. *253*, 155009.

41. Whyte, D., Fisher, S.L., McKenzie, C.G.J., Sumpton, D., Dhayade, S., Dornier, E., Moore, M., Novo, D., Peters, J., Wiesheu, R., et al. (2025). Uridine phosphorylase-1 supports metastasis by altering immune and extracellular matrix landscapes. EMBO Rep. *26*, 4248–4282.

42. Zou, Q., Jiao, J., Zou, M.H., Li, M.Z., Yang, T., Xu, L., and Zhang, Y. (2020). Semi-automatic evaluation of baseline whole-body tumor burden as an imaging biomarker of 68Ga-PSMA-11 PET/CT in newly diagnosed prostate cancer. Abdom. Radiol. *45*, 4202–4213.

43. Rajendran, R., Lap, C.J., Madapoosi, S., Heiraty, A., Estephan, F., Hahn, W., Poudel, A., Nava, V.E., Subrahmanyam, R., and Jain, M. (2025). Clinical Response to Novel Combination of Trastuzumab Deruxtecan and Abiraterone in HER2-Expressing Metastatic Castration-Resistant Prostate Cancer. Oncologist *30*, oyaf207. https://doi.org/10.1093/oncolo/oyaf207.

44. Buteau, J.P., Kostos, L.K., Jackson, P., McIntosh, L., Alipour, R., Akhurst, T.J., Eifer, M., Emmerson, B., Haskali, M.B., Jewell, K., et al. (2025). First-in-human results of terbium-161[ $^{161}$ Tb]Tb-PSMA-I&T radioligand treatment in patients with metastatic castration-resistant prostate cancer (VIOLET): A single-centre, single-arm, phase I/II study. J. Clin. Oncol. *43*, 5010.

45. Bouwman, P., and Jonkers, J. (2012). The effects of deregulated DNA damage signalling on cancer chemotherapy response and resistance. Nat. Rev. Cancer *12*, 587–598.

46. el-Deiry, W.S. (1998). p21/p53, cellular growth control and genomic integrity. Curr. Top. Microbiol. Immunol. *227*, 121–137.

47. Knudsen, E.S., and Knudsen, K.E. (2008). Tailoring to RB: tumour suppressor status and therapeutic response. Nat. Rev. Cancer *8*, 714–724.

48. Chapman, J.D. (2003). Single-hit mechanism of tumour cell killing by radiation. Int. J. Radiat. Biol. *79*, 71–81.

49. Pawlik, T.M., and Keyomarsi, K. (2004). Role of cell cycle in mediating sensitivity to radiotherapy. Int. J. Radiat. Oncol. Biol. Phys. *59*, 928–942.

50. George, J., Lim, J.S., Jang, S.J., Cun, Y., Ozretić, L., Kong, G., Leenders, F., Lu, X., Fernández-Cuesta, L., Bosco, G., et al. (2015). Comprehensive genomic profiles of small cell lung cancer. Nature *524*, 47–53.

51. Beltran, H., Prandi, D., Mosquera, J.M., Benelli, M., Puca, L., Cyrta, J., Marotz, C., Giannopoulou, E., Chakravarthi, B.V.S.K., Varambally, S., et al. (2016). Divergent clonal evolution of castration-resistant neuroendocrine prostate cancer. Nat. Med. *22*, 298–305.

52. Demichev, V., Messner, C.B., Vernardis, S.I., Lilley, K.S., and Ralser, M. (2020). DIA-NN: neural networks and interference correction enable deep proteome coverage in high throughput. Nat. Methods *17*, 41–44.

53. Sychev, Z.E., Day, A., Bergom, H.E., Larson, G., Ali, A., Ludwig, M., Boytim, E., Coleman, I., Corey, E., Plymate, S.R., et al. (2024). Unraveling the Global Proteome and Phosphoproteome of Prostate Cancer Patient-Derived Xenografts. Mol. Cancer Res. *22*, 452–464.

54. Sinha, A., Huang, V., Livingstone, J., Wang, J., Fox, N.S., Kurganovs, N., Ignatchenko, V., Fritsch, K., Donmez, N., Heisler, L.E., et al. (2019). The Proteogenomic Landscape of Curable Prostate Cancer. Cancer Cell *35*, 414–427.e6.

55. Iglesias-Gato, D., Thysell, E., Tyanova, S., Crnalic, S., Santos, A., Lima, T. S., Geiger, T., Cox, J., Widmark, A., Bergh, A., et al. (2018). The Proteome of Prostate Cancer Bone Metastasis Reveals Heterogeneity with Prognostic Implications. Clin. Cancer Res. *24*, 5433–5444.

56. Ludwig, M.L., Arafa, A.T., Vinoo, S., Jones, J.C., Day, A., Bergom, H.E., Sychev, Z., Horrmann, A., Levinson, N.M., Dehm, S.M., et al. (2025). Proteomic profiling of extracellular vesicles distinguishes prostate cancer molecular subtypes. J. Extracell Vesicles *14*, e70176. https://doi.org/10.1002/jev2.70176.

57. Fishilevich, S., Zimmerman, S., Kohn, A., Iny Stein, T., Olender, T., Kolker, E., Safran, M., and Lancet, D. (2016). Genic insights from integrated human proteomics in GeneCards. Database *2016*, baw030.

58. Scher, H.I., Halabi, S., Tannock, I., Morris, M., Sternberg, C.N., Carducci, M.A., Eisenberger, M.A., Higano, C., Bubley, G.J., Dreicer, R., et al. (2008). Design and end points of clinical trials for patients with progressive prostate cancer and castrate levels of testosterone: recommendations of the Prostate Cancer Clinical Trials Working Group. J. Clin. Oncol. *26*, 1148–1159.

59. Subramanian, A., Tamayo, P., Mootha, V.K., Mukherjee, S., Ebert, B.L., Gillette, M.A., Paulovich, A., Pomeroy, S.L., Golub, T.R., Lander, E.S., and Mesirov, J.P. (2005). Gene set enrichment analysis: a knowledge-based approach for interpreting genome-wide expression profiles. Proc. Natl. Acad. Sci. USA *102*, 15545–15550.

## STAR★METHODS

### KEY RESOURCES TABLE

| REAGENT or RESOURCE | SOURCE | IDENTIFIER |
|---|---|---|
| Antibodies | | |
| PSMA antibody | Absolute Antibody | Cat# Ab03136–23.0; RRID: AB_3073514 |
| EpCAM antibody | Biotechne | Cat# BAF960; RRID: AB_356818 |
| Biological samples | | |
| 3mL of plasma for EV isolation | Patient derived | N/A |
| 10 mL of blood | Patient derived | N/A |
| Chemicals, peptides, and recombinant proteins | | |
| PBS (Phosphate-buffered saline) | Sigmaaldrich | Cat# D8537 |
| 2% SDS (Sodium dodecyl sulfate) | sigmaaldrich | Cat# 436143 |
| protease inhibitor | Millipore Sigma | Cat# 11836153001 |
| Phosphatase inhibitor | ThermoFisher | Cat# 78428 |
| LysC | Promega | Cat# VA1170 |
| Trypsin | Worthington | Cat# LS003740 |
| Critical commercial assays | | |
| Pierce™ BCA Protein Assay Kits | Pierce | Cat# PI23227 |
| Pierce quantitative Colorimetric Peptide assay | Pierce | Cat# 23275 |
| Deposited data | | |
| Prostate cancer-focused spectral library | This paper | Ref. 52-55 |
| Proteomics raw files | ProteomeXchange via PRIDE | PXD066814 |
| Software and algorithms | | |
| Syngo.via | Siemens Healthineers | N/A |
| DIA-NN (v2.2) | Cambridge University | N/A |
| R (v4.4.0) | R- project | N/A |
| survival package (v3.6-4) | R- project | RRID: SCR_021137 |
| survminer package (v0.4.9) | R- project | N/A |
| stats package (v4.3.2) | R- project | RRID: SCR_001905 |
| hmisc package (v5.1-1) | R- project | N/A |
| Limma R package | Bioconductor | RRID: SCR_010943 |
| Algorithm for Linking Activity Networks (ALAN) | University of Minnesota | N/A |
| Gene Set Enrichment Analysis (GSEA) | MSigDB | RRID: SCR_003199 |

## EXPERIMENTAL MODEL AND STUDY PARTICIPANT DETAILS

### Study participants

We prospectively enrolled 100 adults with metastatic castration resistant prostate cancer at the University of Minnesota between March 2023 and November 2024. The study was approved by the institutional review board (IRB, number STUDY00013815), and all participants provided written informed consent. Because prostate adenocarcinoma was required for study entry, all patients were male.

The study population consisted of adult male patients with advanced prostate cancer who were clinically fit to receive standard of care $^{177}$Lu-PSMA-617.The median age at study enrollment was 74 years, with a range of 44–94 years. Patients were required to have histologically confirmed prostate adenocarcinoma, progressive disease, at least one PSMA avid metastatic lesion on PSMA PET imaging, and adequate renal function. Patients were excluded if they had another concurrent active malignancy, predominantly PSMA negative metastatic lesions on PSMA PET imaging, PSMA negative liver or brain metastases, histologic evidence of pure neuroendocrine or small cell prostate cancer, or inadequate renal function serum creatinine (<1.5x upper limit of normal). Immune status was

CellPress
OPEN ACCESS

not directly assessed as part of the study and was not used as a stratification variable or formal eligibility criterion. Of 123 patients screened for the study, 23 were excluded due to not meeting all eligibility criteria, resulting in 100 patients enrolled for subsequent biomarker analyses (Figure 1; CONSORT diagram).

Upon consent, patients provided 50mL of peripheral blood collected in EDTA tubes 15 to 60 min prior to the first $^{177}$Lu-PSMA-617 dose. Additional serial blood samples were collected on-study and at the time of disease progression; analysis of these follow-up samples will form the basis of a future manuscript. Plasma was stored at −80°C for downstream EV isolation, and whole blood was sent for CTC processing within 2 h.

### Cancer free controls

We also enrolled 20 cancer free male controls for comparison. The median age of the control group was 60 years, with a range of 54–75 years. Controls had no known cancer diagnosis at the time of enrollment. Immune status was not formally assessed in this group.

## METHOD DETAILS

### EV isolation

EVs were isolated from 3 mL of plasma using our previously reported ultracentrifugation-based protocol.[19] Briefly, plasma was centrifuged at 10,000 g for 30 min to remove debris, followed by ultracentrifugation at 100,000 g to form EV pellets. EVs were then washed with PBS through four sequential ultracentrifugation cycles at 100,000 g, resuspended in 2% SDS containing protease/phosphatase inhibitors, and quantified by the bicinchoninic acid (BCA) assay. Proteins were reduced, alkylated, and processed using single pot, solid phase enhanced sample preparation (SP3). Peptides were digested with LysC and trypsin (1:50 enzyme to protein ratio) and quantified prior to liquid chromatography-mass spectrometry (LC-MS) analysis, as described below.[19]

### Mass spectrometry

Mass spectrometry data were acquired using an Orbitrap Exploris 480 mass spectrometer coupled to a Vanquish *Neo* nanoflow UPLC with a Nanoflex ion source (Thermo Scientific, Waltham, MA, USA) fitted with a column heater (Sonation GmbH, Model PRSO-V2, Biberach, Germany). Chromatography was performed using an Aurora Frontier (IonOpticks, Fitzroy, Victoria, Australia) Ultra-High Performance Liquid Chromatography (UHPLC) packed emitter C18 column (75 μm ID × 60 cm, 1.7 μm particlesize) maintained at 50°C. The LC solvents were (A) 0.1% formic acid in water and (B) 0.1% formic acid in 80% acetonitrile. Peptides were separated at a flow rate of 250 nL/min using the following LC gradient: 3.8–7.5% buffer B in 2.5 min, 7.5–30% buffer B in 160 min, 30–43.8% buffer B in 10.5 min, 43.8–100% buffer B in 0.5 min, and, finally, 100% buffer B for 6.5 min at 300 nL/min, for a total run time of 180 min. The following ion source parameters were used: spray voltage = 1900 V, ion transfer temperature = 285°C, RF lens = 45%. The data independent acquisition (DIA) tandem mass spectrometry method used the following parameters: precursor ions from 380 to 1200 m/z were scanned at a resolution of 60,000 with a maximum ion injection time of 25 ms and a normalized Automatic Gain Control (AGC) target of 300%. The MS/MS data were acquired using Higher energy Collisional Dissociation (HCD) fragmentation at a collision energy of 28% and covering a precursor scan range from 380 to 980 m/z with 60 DIA windows centered 10 m/z apart with 1 m/z overlap and at a resolution of 15,000, a maximum injection time of 40 ms, a normalized AGC target of 1000%, and a product ion scan range of 145–1450 m/z.

### Processing of mass spectrometry proteomic data

DIA raw files were analyzed using DIA-NN (v2.2, Thermo, Cambridge, UK).[52] Files were converted to.dia format using DIA-NN's built-in tool. A prostate cancer-focused spectral library was generated by integrating datasets from four previously published proteomic studies, yielding 11,972 proteins. Contaminating proteins ($n$ = 114) were removed including immunoglobulins, fibrinogen chains, complement proteins, and keratins, resulting in a final library of 11,858 proteins[53–56] (Figure S1). FASTA files were prepared using trypsin/P digestion, allowing up to two missed cleavages and one variable modification. Peptides were filtered based on a length of 7–30 amino acids, precursor charge of 1–4, and m/z ranges of 300–1800 for precursors and 200–1800 for fragments. Quantification matrices were generated using a 1% false discovery rate (FDR) at the precursor as well as the protein level.

### Nanotracker analysis

The EV samples were analyzed for size and particle concentration using ZetaView Quatt PMX-430 (Particle Metrix GmbH, Inning am Ammersee, Germany) with ZetaView Software Suite (v.1.3). The samples were diluted to manufacturer recommendations of optimal particles per view. The measurements were collected using scatter with a 488 nm laser wavelength, recording at least 9 positions with 30 s of video, a camera rate of 30 frames per second, a set temperature of 25°C, sensitivity of 85, and a shutter speed of 200.

### Protein compartmentalization

The subcellular localization of the proteins was annotated using GeneCards.[57] A hierarchical classification was applied in the following order: cell membrane > nucleus > cytoplasm > mitochondria > other (including endoplasmic reticulum, endosome, peroxisome, Golgi apparatus, and lysosome).

### CTC enumeration

CTC enumeration was conducted as previously described.[27] Briefly, 20 mL of whole blood underwent red blood cell and leukocyte depletion via a microfluidic chip, yielding 6 mL of enriched cells. Cells were labeled with fluorescently-conjugated antibodies targeting PSMA and epithelial cell adhesion molecule (EpCAM), alongside Hoechst nuclear stain. CTCs were analyzed and enumerated using a dual-modality system combining high-throughput digital holographic microscopy and immunofluorescence staining. CTCs were defined as Hoechst-positive cells that exceeded a deep learning-based holographic morphology threshold and expressed PSMA with or without concurrent EpCAM expression. This multimodal platform enabled real-time label-assisted identification of both EpCAM-positive and EpCAM-negative CTC subpopulations with a low false-positive rate (of <1 cell/mL), allowing for high-sensitivity enumeration in patient-derived blood samples.

### Clinical outcomes

After $^{177}$Lu-PSMA-617 initiation, patients were assessed for PSA response, progression-free survival (PFS) and overall survival (OS). PSA measurements were generally obtained every 6 weeks ( ±2 weeks), and imaging assessments (CT, bone scans) were generally obtained every 12 weeks ( ±4 weeks). PSA50 response was defined as a decline in PSA of at least 50% from baseline confirmed by a second measurement at least 3 weeks later. PFS was defined as the time from initiation of $^{177}$Lu-PSMA-617 therapy to the first documented occurrence of disease progression or death from any cause. Disease progression was determined by one or more of the following: biochemical progression (defined as two consecutive rises in PSA levels obtained at least 3 weeks apart), radiographic progression (on conventional imaging), or clinical progression resulting in treatment discontinuation due to worsening symptoms.[58] Patients who did not meet any of these criteria were censored at the date of their last follow-up. Overall survival (OS) was defined as the time from initiation of therapy to death from any cause. Patients who were alive at the time of data cutoff were censored at the date of last follow-up.

### PSMA-PET imaging

All patients were required to undergo baseline standard-of-care PSMA-PET/CT imaging (not provided by the study) using any of the FDA-approved PSMA molecular imaging agents. PSMA-PET/CT images were analyzed using a dedicated workstation equipped with a commercial software package (Syngo.via; Siemens Healthineers, Erlangen, Germany). Focal radiotracer uptake higher than liver background in the suspected areas were accepted as malignant lesions. SUVmax and SUVmean were measured in all volumes of interest using this software. Molecular Tumor Volume (MTV) burden was defined as the sum of all PSMA receptor expression positive tumor identified in the body on a PSMA PET. To calculate volumetric parameters i.e., MTV, an isocontour volume of interest including all voxels above 40% of the maximum was created using a three-dimensional segmentation technique.

## QUANTIFICATION AND STATISTICAL ANALYSIS

### Biostatistics analysis

Statistical analyses were performed using R version 4.4.0. We dichotomized the transformed CTC/EV protein into two groups based on detection rates. Proteins with a detection rate above 80% were categorized as above versus below median, whereas proteins with a detection rate below 80% were dichotomized into detected vs. undetected groups. Cox proportional hazards models fitted using the coxph function in R to assess the relationship between binary CTC/EV protein groups (1) without adjusting for any covariates and PFS/OS. (2) with adjustments for baseline serum PSA level, baseline hemoglobin (Hb), and baseline alkaline phosphatase (ALP). PSMA PET parameters, including SUVmean, SUVmax, and MTV burden, were dichotomized into binary variables based on median levels. Multivariable logistic regression was performed for binary PSA50 response and the same set of covariates were adjusted. Odds ratios (OR) or Hazard ratios (HR), *p*-values from Wald tests, and 95% confidence intervals (CI) were reported. Spearman's rank correlation (r) was used for nonparametric correlation analyses, while $R^2$ values were calculated from squared Pearson correlation results to quantify the amount of unexplained variance due to a linear relationship. Mann Whitney U-tests were used to robustly compare differences in distribution between two independent groups. Kruskal-Wallis tests were used to robustly compare differences in distribution across three or more independent groups. Kaplan-Meier (K-M) survival curves, forest plots, and scatterplots were generated using the survival package (version 3.6–4), specifically the coxph() and survfit() functions, and visualized using survminer (version 0.4.9). For correlation analyses, non-parametric Spearman's correlation coefficients were calculated using the stats package (version 4.3.2) and visualized using Hmisc (version 5.2–4).

### Prognostic modeling and internal validation

We evaluated two sets of prognostic modeling. In the first set, we performed univariable and multivariable regression models using different combinations of the 4 EV protein or CTC groups and other clinical factors to assess the prognostic impact in predicting PSA50 response or PFS/OS. Variables were log2 transformed where appropriate, and continuous predictors were modeled using restricted cubic splines via the rcs() function from the rms R package (version 8.1–0) when needed after being assessed for potential nonlinearity. In the second set, we performed an agnostic search of EV proteins from 1600 proteins not found in healthy control patients. All EV proteins were analyzed as binary variables as described above, and only proteins with a detection rate of at least 10% were considered for modeling. Feature selection was performed using LASSO regression as implemented in the glmnet R package

CellPress
OPEN ACCESS

(version 4.1–10). For the survival models, type.measure = "C" and family = "cox" was used with 10-fold cross-validation and the regularization parameter was selected by maximizing the Harrell's concordance index. Category-free net reclassification improvement (NRI) was calculated at 6 and 12 months for PFS and OS respectively using the nricens package (version 1.6). For the binary PSA50 models, we used glmnet package and set type.measure = "auc" and family = "binomial" with 5-fold cross-validation. The regularization parameter was selected by maximizing the area under the ROC curve. Category-free NRI was calculated using the improvedProb function from the Hmisc package. For both types of models, only the top 9 protein variables with the largest absolute non-zero coefficients selected at lamda.min were then retained with either MTV burden or PSA in a standard Cox proportional hazards or logistic regression model for further internal validation.

We report the optimism-corrected C-indices (survival models), AUC scores (PSA50 models), and NRI values using Harrell's bootstrap optimism method with 200 bootstrap resamples (B = 200). Optimism-corrected calibration metrics were estimated using the rms R package with 200 bootstrap resamples. For each resample, each given model was refitted (only top 9 protein variables from the second set were used) and evaluated on both the bootstrap and original samples. The prediction performance metrics were then corrected through subtracting the average optimism from the apparent performance of each metrics. Feature selection stability was assessed using 200 bootstrap resamples by recording the proportion of resamples in which each feature was selected by LASSO as described previously and/or ranked among the top 9 by coefficient magnitude.

## Differential protein expression

DIANN MaxLFQ-normalized data were further normalized by dividing by the total EV counts for each patient, and were subsequently log2-transformed. Groups of samples were annotated by their detection/expression status of EV-derived PSMA, B7-H3, Trop-2, and STEAP1 protein detection and concentration based on this data. The aggregate protein expression profiles from both groups were analyzed using the Limma R package, which reports the log2 fold change and statistics for each of the proteins based on two annotated groups of samples.To account for mean-variance relationships, Empirical Bayes moderation of the standard errors was applied with robust variance estimation and a trend prior (trend = TRUE, robust = TRUE).

## GSEA

Pre-ranked and single-sample GSEA (ssGSEA) analyses were performed on the MaxLFQ abundance levels of all proteins to assess the enrichment of the Hallmark pathways from MSigDB between certain groups of interest as well as across all patients in general.[59] For pre-ranked GSEA studies, we established ranked association for key targets PSMA, B7-H3, Trop-2, and STEAP1 proteins based on their association with all detectable proteins. Briefly, the samples were first grouped based on high- and low-expression status or detection status based on all 100 samples. The DEP output from Limma as described above was then used to generate the input for the pre-ranked profile based on multiplying the signage of the log2 fold change by the -log10 ($p$-value). For ssGSEA, we directly used the ranked expression of all proteins as input for each sample. For both analyses, a minimum gene set size of 15 was used in order to account for potential low detection rates for certain Hallmark gene sets.

## ALAN

The expression of proteins was processed and analyzed using ALAN, in which all pairwise interactions between the detectable proteins were compared and assigned values between $-1$ and 1, which indicates relative similarity of networks.[36] Briefly, we first generated a data matrix for all proteins and all samples using the MaxLFQ-normalized and log2 transformed data. Internally, pairwise correlations were calculated using a threshold of 15 complete observations, such that if a given correlation between two proteins was calculated using less than 15 samples with non-NA data, the resulting correlation would be deemed NA. The ALAN profiles of PSMA, B7-H3, Trop-2, and STEAP1 proteins were then analyzed and presented based on our thresholds previously reported.[36]

Cell Reports Medicine, Volume *7*

## Supplemental information

# Plasma extracellular vesicle proteomics nominates candidate biomarkers of $^{177}$Lu-PSMA-617 outcomes in metastatic prostate cancer patients

**Ali T. Arafa, Ella Boytim, Megan L. Ludwig, Lily Kollitz, Tianzhong Yang, Kathleen M. Storey, Stuart Bloom, Gautam Jha, Ian Okazaki, Charles J. Ryan, Nicholas A. Zorko, Daniel Steinberger, Zuzan Cayci, Yingchun Zhao, Peter W. Villalta, Scott M. Dehm, Justin H. Hwang, Justin M. Drake, and Emmanuel S. Antonarakis**

# Supplemental Figure 1

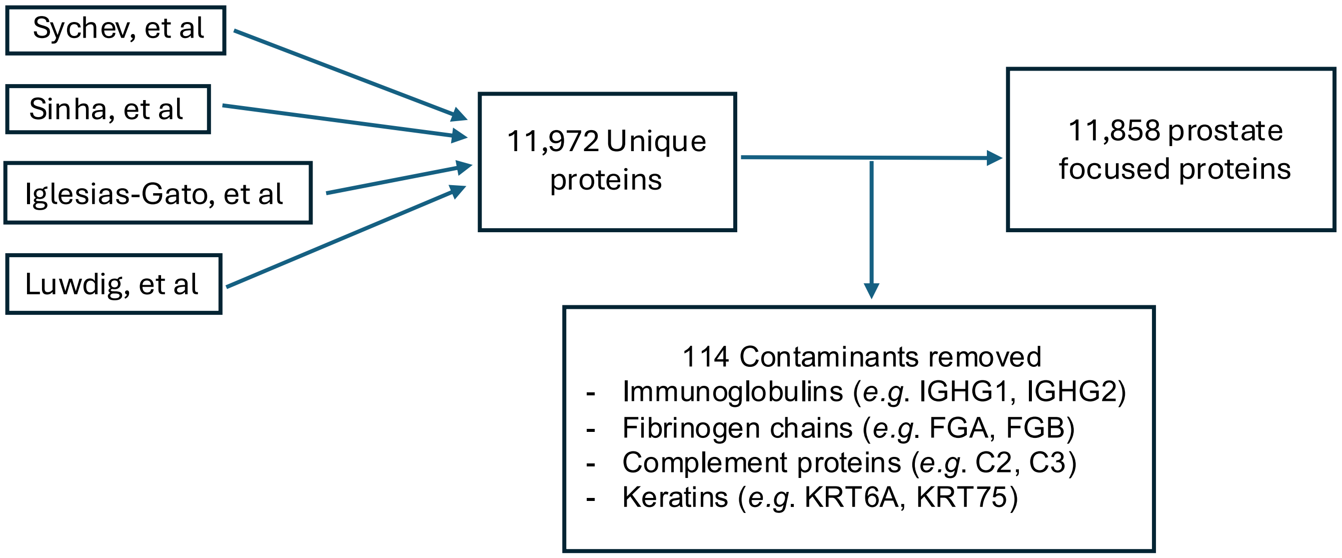


**Supplemental Figure 1. Consort-like diagram summarizing how the EV proteomic library was created.** A total of 11,972 proteins were identified by integrating prostatic cancer proteomic datasets from four independent studies: Sychev et al., 2024, Sinha et al., 2019, Iglesias-Gato et al., 2018, and Ludwig et al., 2025. After removing 114 contaminants, including immunoglobulins (e.g., IGHG1, IGHG2), fibrinogen chains (e.g., FGA, FGB, FGG), complement proteins (e.g., C2, C3), and keratins (e.g., KRT8, KRT17), a total of 11,858 prostate focused proteins were retained for the final EV proteomic library.

# Supplemental Figure 2

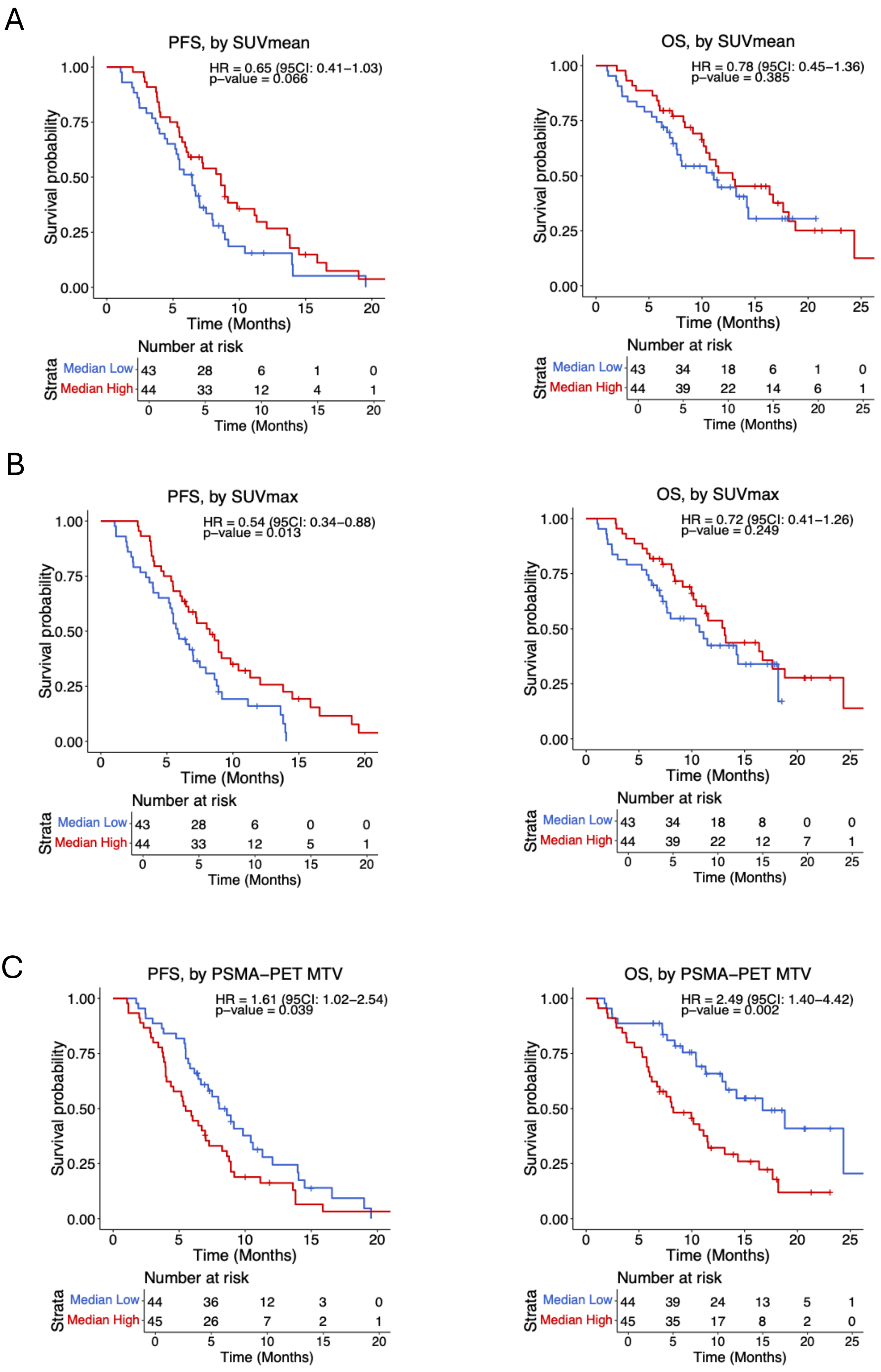


**Supplemental Figure 2: Prognostic value of PSMA-PET imaging parameters in patients undergoing $^{177}$Lu-PSMA-617 therapy.** A. Kaplan-Meier curves showing no significant association between SUVmean and PFS (left) or OS (right). B. Kaplan-Meier curves showing that higher SUVmax is significantly associated with improved PFS (left) but not OS (right). C. Kaplan-Meier analysis of molecular tumor volume (MTV) burden showing that patients with high MTV had worse PFS (left) or OS (right).

Supplemental Figure 3

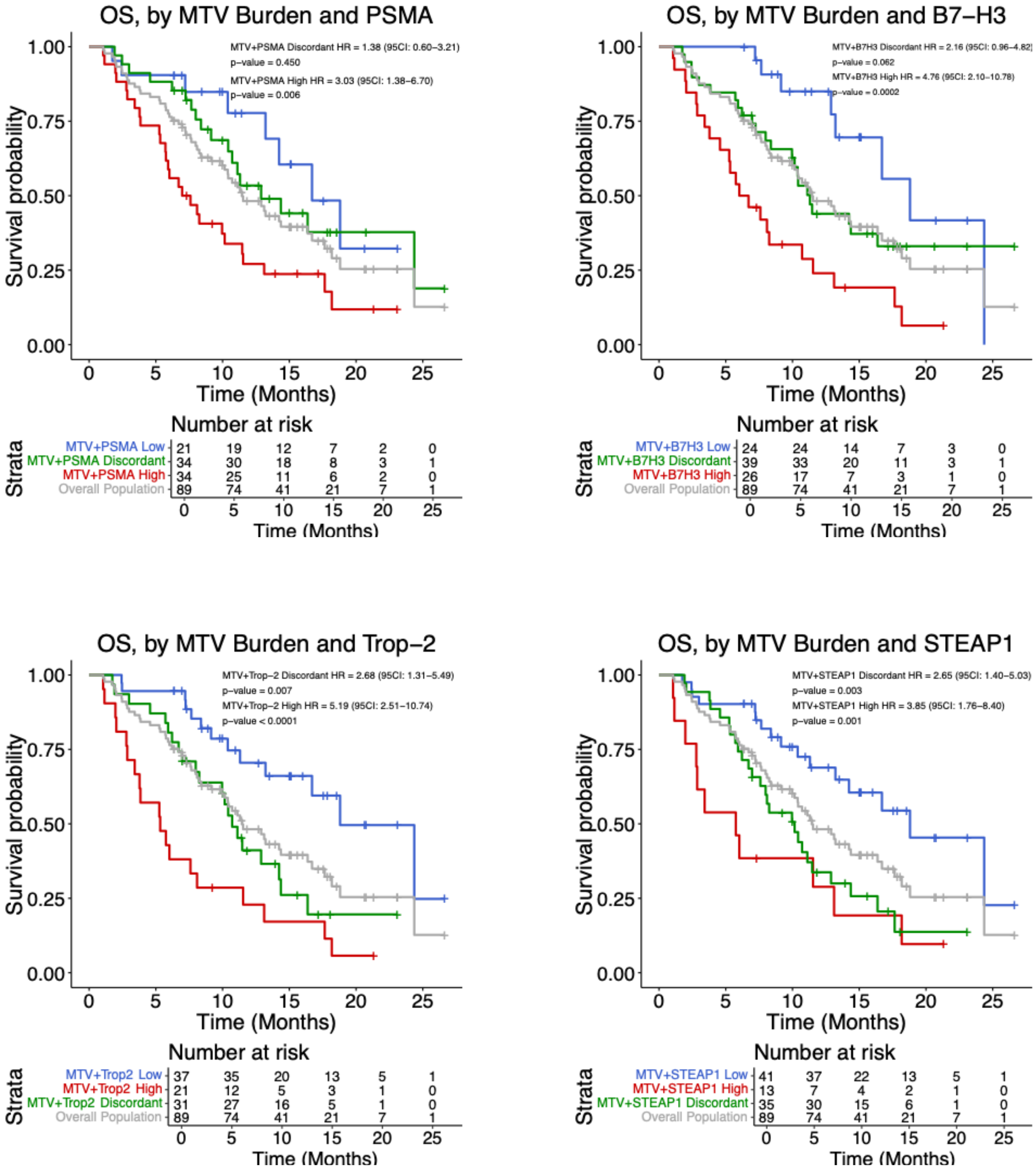


**Supplemental Figure 3: Prognostic utility of EV surface proteins beyond MTV burden.** A. Kaplan-Meier curve showing that MTV burden + PSMA high levels were significantly associated with worse overall survival (OS). B. Kaplan-Meier curve showing that MTV burden + B7-H3 high levels were significantly associated with worse overall survival (OS). C. Kaplan-Meier curve showing that MTV burden + Trop-2 high levels were significantly associated with worse overall survival (OS). D. Kaplan-Meier curve showing that MTV burden + STEAP1 high levels were significantly associated with worse overall survival (OS).

# Supplemental Figure 4

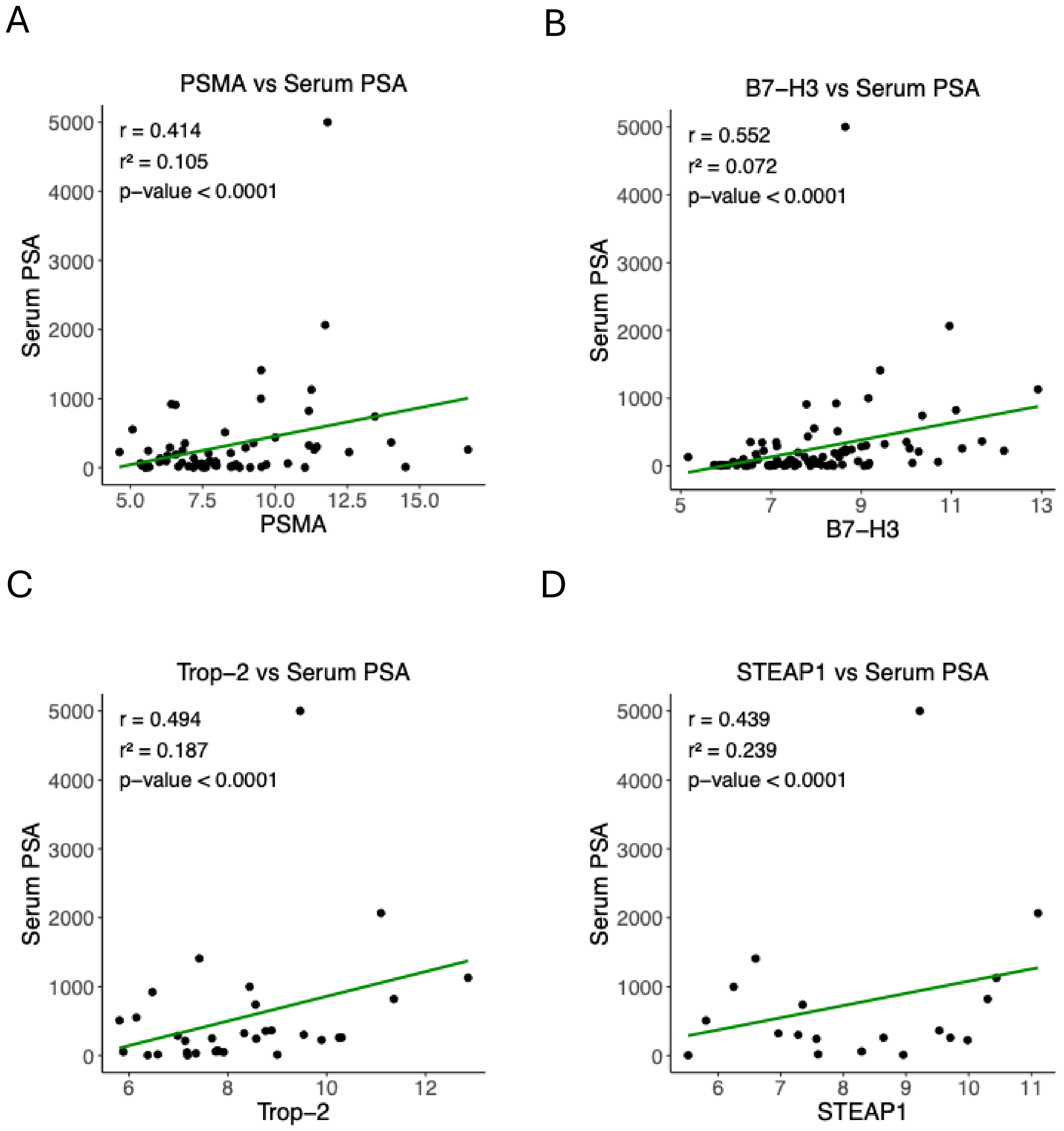


**Supplemental Figure 4. Correlation between EV surface proteins and serum PSA levels.** A. Correlation between serum PSA levels (y-axis) and EV-derived PSMA protein (x-axis). B. Correlation between serum PSA levels (y-axis) and EV-derived B7H3 protein (x-axis). C. Correlation between serum PSA levels (y-axis) and EV-derived Trop-2 protein (x-axis). D. Correlation between serum PSA levels (y-axis) and EV-derived STEAP1 protein (x-axis).

# Supplemental Figure 5

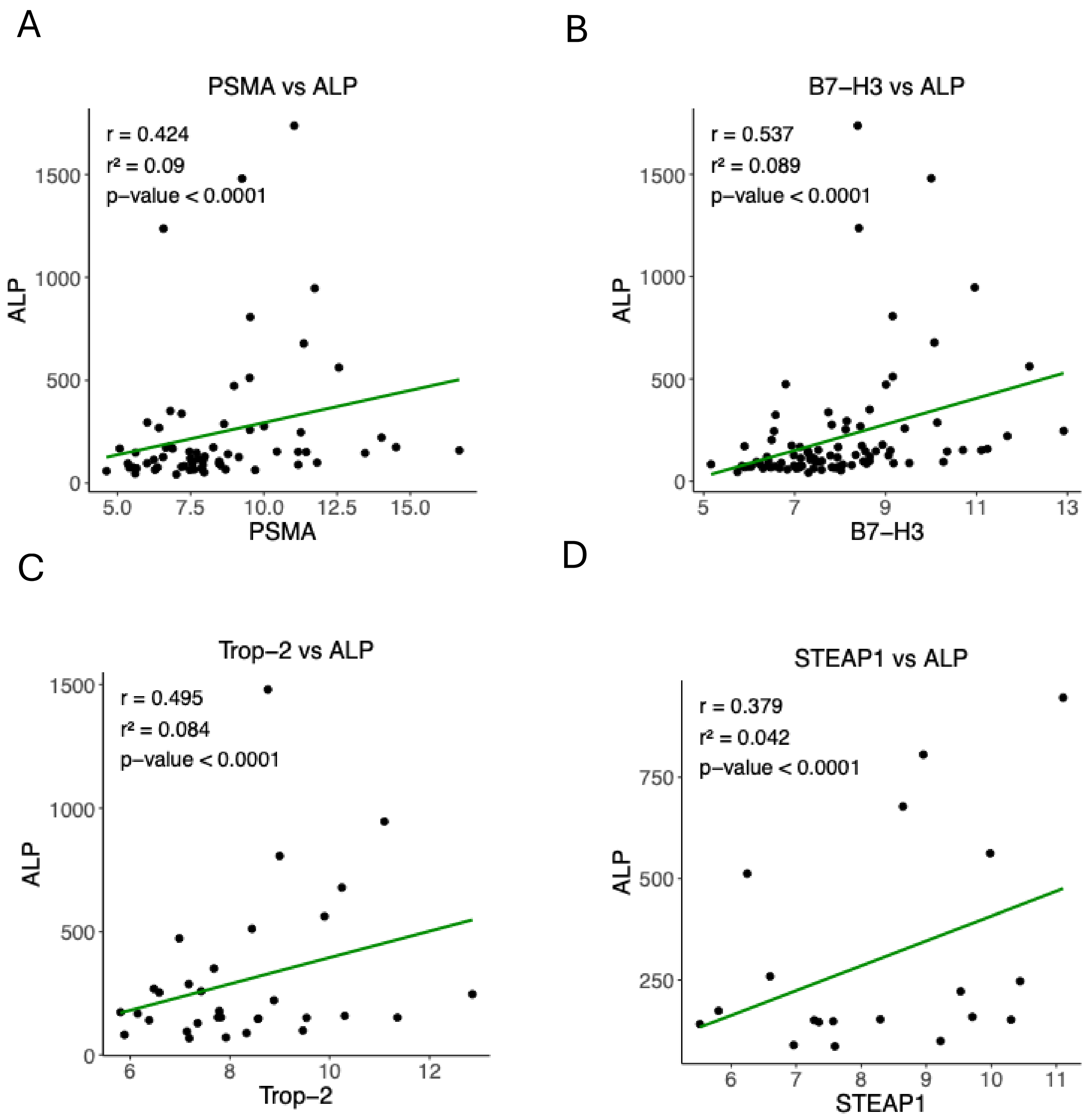


**Supplemental Figure 5. Correlation between EV surface proteins and serum ALP levels.** A. Correlation between serum ALP levels (y-axis) and EV-derived PSMA protein (x-axis). B. Correlation between serum ALP levels (y-axis) and EV-derived B7H3 protein (x-axis). C. Correlation between serum ALP levels (y-axis) and EV-derived Trop-2 protein (x-axis). D. Correlation between serum ALP levels(y-axis) and EV-derived STEAP1 protein (x-axis).

Supplemental Figure 6

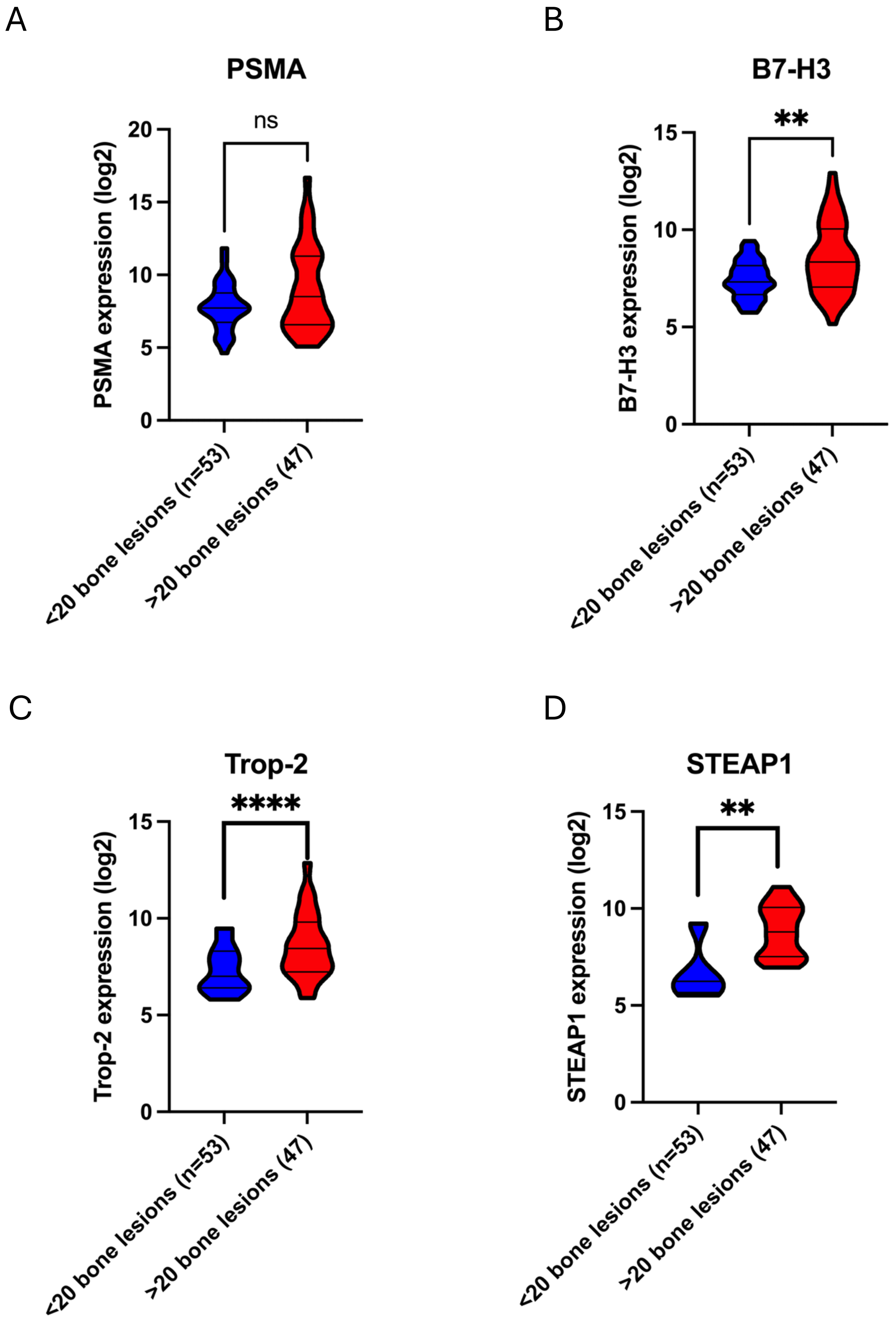


**Supplemental Figure 6. EV protein expression association with bone metastatic burden.** Violin plots comparing EV surface protein expression between patients with <20 vs >20 bone metastases with respect to A. PSMA protein, B. B7-H3 protein, C. Trop-2 protein, and D. STEAP1 protein. Plots display median expression levels, with x-axis indicating bone metastasis groups and y-axis representing $\log_{10}$-transformed protein expression values. Asterisk marks denote level of statistical significance (Mann Whitney U-tests) : $*p < 0.05$, $**p < 0.01$, $***p < 0.001$, $****p < 0.0001$.
